# Small RNA-Directed Epigenetic Natural Variation in *Arabidopsis thaliana*

**DOI:** 10.1371/journal.pgen.1000056

**Published:** 2008-04-25

**Authors:** Jixian Zhai, Jun Liu, Bin Liu, Pingchuan Li, Blake C. Meyers, Xuemei Chen, Xiaofeng Cao

**Affiliations:** 1 State Key Laboratory of Plant Genomics, Institute of Genetics and Developmental Biology, Chinese Academy of Sciences, Beijing, China; 2 National Center for Plant Gene Research, Institute of Genetics and Developmental Biology, Chinese Academy of Sciences, Beijing, China; 3 Graduate University of the Chinese Academy of Sciences, Beijing, China; 4 Department of Botany and Plant Sciences, Institute of Integrative Genome Research, University of California Riverside, Riverside, California, United States of America; 5 Delaware Biotechnology Institute, University of Delaware, Newark, Delaware, United States of America; 6 Department of Plant and Soil Sciences, University of Delaware, Newark, Delaware, United States of America; The Salk Institute for Biological Studies, United States of America

## Abstract

Progress in epigenetics has revealed mechanisms that can heritably regulate gene function independent of genetic alterations. Nevertheless, little is known about the role of epigenetics in evolution. This is due in part to scant data on epigenetic variation among natural populations. In plants, small interfering RNA (siRNA) is involved in both the initiation and maintenance of gene silencing by directing DNA methylation and/or histone methylation. Here, we report that, in the model plant *Arabidopsis thaliana*, a cluster of ∼24 nt siRNAs found at high levels in the ecotype Landsberg *erecta* (L*er*) could direct DNA methylation and heterochromatinization at a *hAT* element adjacent to the promoter of *FLOWERING LOCUS C* (*FLC*), a major repressor of flowering, whereas the same *hAT* element in ecotype Columbia (Col) with almost identical DNA sequence, generates a set of low abundance siRNAs that do not direct these activities. We have called this *hAT* element *MPF* for *Methylated region near Promoter of FLC*, although *de novo* methylation triggered by an inverted repeat transgene at this region in Col does not alter its *FLC* expression. DNA methylation of the L*er* allele *MPF* is dependent on genes in known silencing pathways, and such methylation is transmissible to Col by genetic crosses, although with varying degrees of penetrance. A genome-wide comparison of L*er* and Col small RNAs identified at least 68 loci matched by a significant level of ∼24 nt siRNAs present specifically in L*er* but not Col, where nearly half of the loci are related to repeat or TE sequences. Methylation analysis revealed that 88% of the examined loci (37 out of 42) were specifically methylated in L*er* but not Col, suggesting that small RNA can direct epigenetic differences between two closely related Arabidopsis ecotypes.

## Author Summary

Phenotypic variation has been mainly attributed to their differences in genetic materials, i.e., the DNA sequence. The advances in Epigenetics in past decades has revealed it as a fundamental mechanism that could inheritably influence gene function without change in DNA sequence, but by modulating chemical modifications on DNA itself (methylation), or on histone proteins, which package the DNA further into nucleosome. Nevertheless, the roles of epigenetic regulation in natural variation were not explored much because of the limitation in high-throughput analytical tools. A recent study in model plant Arabidopsis showed that there are many DNA methylation polymorphisms between the two ecotypes. In plant, a subset of RNA named small interfering RNA (siRNA), is capable of triggering the epigenetic modifications on DNA or histone at their target region with complementary nucleotide sequences. Here, we took a view from the small RNA side and by applying molecular and bioinformatic approaches we showed that the same region could be led to a different epigenetic status because of the difference in their corresponding small RNA abundance and between the two closely related Arabidopsis ecotypes, suggesting that there could be small RNA-directed epigenetic differences among natural populations.

## Introduction

Epigenetics, defined as the study of heritable alteration in gene expression without changes in DNA sequence, has greatly expanded our understanding of inheritance [1]. A recent study of DNA methylation by tiling array analysis of *Arabidopsis* Chromosome 4 in Col and L*er* showed that although transposable elements (TEs) are often methylated, the methylation in the transcribed regions of genes is highly polymorphic between these two ecotypes [2]. Although epigenetic differences could potentially contribute to evolution [3]–[5], studies of evolution and natural variation have still been focused mainly on sequence variation, and little is known about the role of epigenetic machinery in these processes. This is primarily due to the lack of evidence for epigenetic natural variation between populations.

Small interfering RNAs (siRNAs), as a key player in the epigenetic machinery, have been well documented for their general role in gene silencing at both the transcriptional and post-transcriptional levels [6],[7]. In *Arabidopsis*, ∼24 nt siRNAs can direct DNA methylation (RNA-directed DNA methylation, RdDM) and chromatin remodeling at their target loci [8]. In the RdDM process, ∼24 nt siRNAs are incorporated into ARGONAUTE 4 (AGO4)-containing complexes and further guide the DOMAINS REARRANGED METHYLTRANSFERASE 2 (DRM2) to *de novo* methylate their target DNA [9],[10]; once established, the non-CG methylation could be maintained by DRM2 and/or CHROMOMETHYLASE 3 (CMT3) in a locus-specific manner, and the CG methylation by METHYLTRANSFERASE 1 (MET1) [11]. Recent advances in high-throughput sequencing techniques have enabled the thorough exploration of the small RNAs populations [12]–[16]. Therefore, together with the complete genome sequence, we are able to directly examine whether there are regions specifically matched by siRNAs that differ among ecotypes, a situation that could lead to epigenetic natural variation.

*FLC*, a MADS box transcription factor, is a major repressor of the transition to flowering in *Arabidopsis*, and many genes coordinately function in flowering time control by regulating the amount of *FLC* transcript [17]. In addition, allelic variation at *FLC*, both genetic [18]–[21] and epigenetic [22],[23], contributes to the differences in flowering time and vernalization response among accessions, which makes *FLC* a classic locus for the study of natural variation in *Arabidopsis*. Previous studies have shown that in L*er*, a 1224 base pair (bp) nonautonomous *Mutator*-like transposable element (TE) inserted in the first intron of *FLC* (*FLC*-TE-L*er*) [19] was methylated and heterochromatic under the direction of ∼24 nt siRNAs generated by homologous TEs, and mutation of *HUA ENHANCER 1 (HEN1)* in L*er* (*hen1-1*), a key component in small RNA biogenesis [7], released the transcriptional silencing of *FLC*-L*er* [22].

In this study, we discovered a cluster of ∼24 nt siRNAs that are present at high levels in the ecotype L*er* and that could direct DNA methylation and heterochromatinization adjacent to *FLC* promoter [24]. However siRNAs matching to the same region in Col are of low abundance and cannot direct DNA methylation. Furthermore, from comparisons between L*er* and Col of small RNA data produced by high-throughput sequencing, we identified at least 68 loci that are matched by significant levels of ∼24 nt siRNAs, and 88% are methylated in L*er* but not Col from a set of 42 loci that were examined.. Although siRNA clusters are often heavily methylated [25] and a large proportion of the methylation polymorphisms between Col and L*er* are not associated with small RNAs [2], our data reveal that there could still be considerable small RNA-directed epigenetic natural variation between two ecotypes of *Arabidopsis*.

## Results

### A Region Adjacent to the Promoter of *FLC* is Methylated in L*er* but not Col

**Figure 1:**
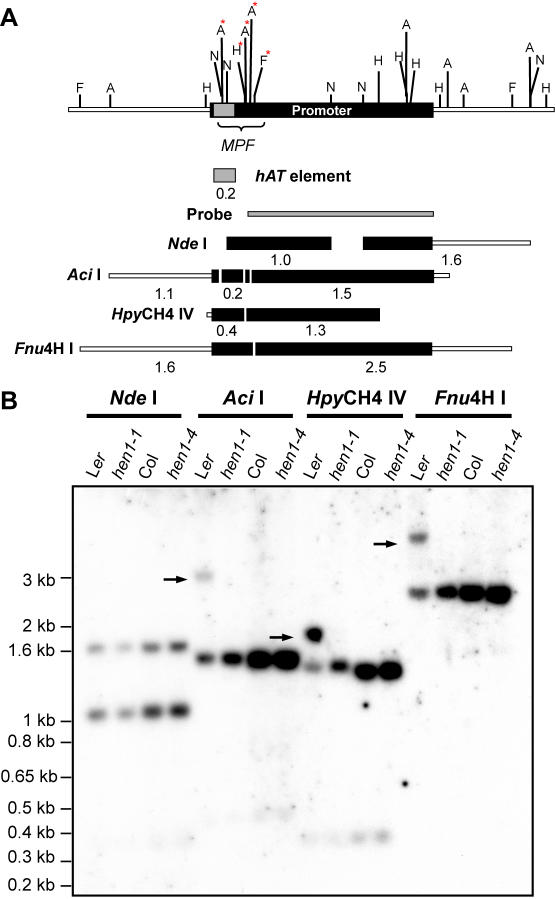
DNA Methylation Analysis of the *FLC* Promoter by Southern Blots. (A) A diagram of the genomic region around *FLC* promoter is shown above with the positions of restriction sites marked as follows: *Fnu*4H I (F), *Aci* I (A), and *Hpy*CH4 IV (H) are sensitive to methylation; *Nde* I (N) which is not sensitive to methylation is used as a negative control. Red stars highlight the methylated sites. The digested fragments that could be detected by probe covering *FLC* promoter (gray strip) were diagramed and the size is indicated by numbers (in kilobases) beneath the fragments. A *hAT* element is represented as gray box (see Figure 4 for more detail). (B) Determination of DNA methylation status at *FLC* promoter in L*er*, *hen1-1*, Col, and *hen1-4*. Black arrows indicate the DNA fragments which contain the methylated (and therefore uncut) enzyme recognition sites.

**Figure 2:**
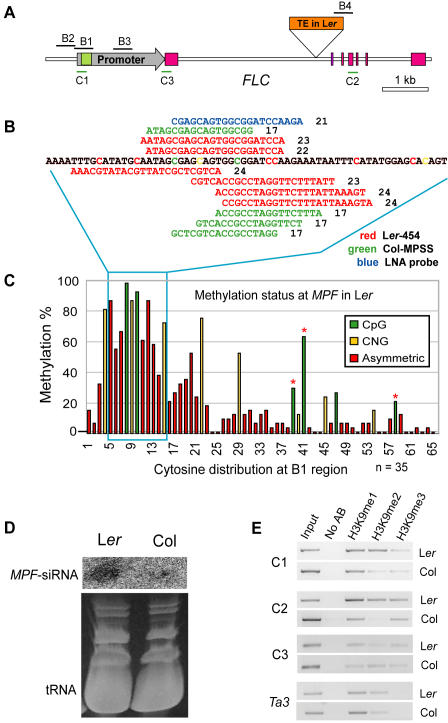
RNA-directed DNA Methylation and Heterochromatinization at the *MPF.* (A) Genomic structure of the *FLC* locus and flanking regions examined by bisulfite sequencing (B1, B2, B3 and B4) or ChIP (C1, C2 and C3). Green box represents the *hAT* element; pink boxes represent exons; the gray arrow represents the promoter; the orange box represents the TE insertion in L*er*. (B) Small RNA tags matched to *MPF* found from the MPSS (green) or 454 sequencing data (red), and the LNA probe used for small RNA hybridization (blue) are represented with their length indicated by numbers. The color coding of the cytosines in (B) matches the legend in (C). (C) Bisulfite sequencing result of the *MPF* at the B1 region in L*er*. The bars with red stars represent sites that were detected by Southern blot (Figure 1) and n indicates the number of the sequenced clones. (D) Small RNA Northern blots probed with the LNA probe (B) in L*er* and Col; tRNA and other RNA bands stained with ethidium bromide (EtBr) were used to indicate the amount of loaded RNA. (E) Chromatin Immunoprecipitation (ChIP) to detect H3K9 mono-, di-, and tri-methylation (represented as H3K9me1, H3K9me2, and H3K9me3, respectively) at *MPF* (C1) in L*er* and Col. Input is saved before immunoprecipitation and “No AB” refers to the sample without antibody. *Ta3* served as an internal control for heterochromatic loci.

**Figure 3:**
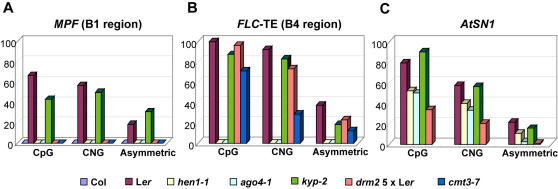
Methylation Analysis of *MPF* and *FLC-*TE. Bisulfite sequencing analysis of DNA methylation at the *MPF* (A), *FLC*-TE (B) and *AtSN1* (C) in Col, L*er*, *hen1-1*, *ago4-1*, *kyp-2*, *drm2* 5×L*er*, and *cmt3-7*, summarized in different sequence contexts. Methylation status had been independently confirmed by bisulfite sequencing or McrBC PCR for at least four times.

In addition to the previously described *Mutator*-like transposable element (TE) inserted in the first intron of *FLC* [19] in L*er*, we found that a region located adjacent to the promoter of the *FLC* was specifically methylated in L*er* but not in Col (Figure 1A). We named this region *MPF* (*Methylated region near Promoter of FLC*). Restriction enzymes including *Aci*I, *Hpy*CH4 IV and *Fnu*4HI, which are sensitive to CpG methylation, were able to cut outside of the *MPF* but not within this region in L*er* (Figure 1). Notably different from the TE inserted in *FLC*-L*er*, the *MPF* of L*er* and Col share almost identical sequences (Figure S1). Bisulfite sequencing of *MPF* (B1 region, Figure 2A) revealed that a small region of less than 100 bp was exhibited a very high level of asymmetric methylation (also called CHH methylation, where H represents A, C or T) (Figure 2C). This region also demonstrated extensive CpG and CNG (where N is any nucleotide) methylation (Figure 2C). In addition, no DNA methylation was found outside the *MPF* (the B2 and B3 regions, Figure 2A) in L*er* (data not shown) or the *MPF* in Col (Figure 3A) by bisulfite sequencing.

### High Levels of *MPF*-siRNAs in L*er*, but not Low Levels in Col, Direct DNA Methylation and Heterochromatinization at *MPF*

Since asymmetric methylation is the hallmark of RdDM [26], we decided to verify whether there are corresponding siRNAs matching to this methylated region in L*er*. Because no methylation was found at the *MPF* in Col, we speculated that there would be no small RNAs matching to this region. However, four 17 nt tags with very low abundances (approximately two transcripts per quarter-million, TPQ) were found in the Col-derived small RNA massively parallel signature sequencing (MPSS) datasets [12]. These small RNAs precisely matched both strands of the highly asymmetrically methylated region within *MPF* (Figure 2B). We performed a small RNA Northern blot hybridization to verify these small RNA in Col and L*er*. By using an LNA (locked nucleic acid) modified oligonucleotide probe (Figure 2B) and a large amounts of RNA enriched for small RNAs (see materials and method for more details), we found that siRNAs complementary to this probe (*MPF*-siRNAs) were more abundant in L*er* than in Col (Figure 2D). Published high-throughput small RNA 454 sequencing datasets from L*er* [15] confirmed our RNA gel blot results. In those data, six unique 23 to 24 nt small RNAs were found matching to a region of <50 bp at the *MPF*, in exactly the same region as the Col-derived *MPF*-siRNAs (Figure 2B). Analyses of additional Col-derived 454 small RNA data [16],[27] didn't identify any *MPF*-matching small RNAs, possibly due to lower sequencing depth compared to that of the MPSS data. We performed chromatin immunoprecipitation (ChIP) experiments and demonstrated that the *MPF* in L*er* was enriched in H3K9me2, a characteristic of heterochromatin, in comparison to Col (Figure 2E). These data suggest that the high levels of *MPF*-siRNAs in L*er* could trigger DNA methylation and heterochromatinization at *MPF* whereas the lower levels in Col might not be sufficient.

### Methylation at *MPF* Is Sensitive to Deficiency in RdDM

Next, we investigated methylation at the *MPF* using silencing pathway mutants in either a L*er* background or in lines that had been backcrossed to L*er* to have the homozygote *FLC*-L*er* allele. These mutants included *hen1-1*, *cmt3-7*, *ago4-1*, *kryptonite-2* (*kyp*, a histone H3K9 methyltransferase, also known as SUVH4, can affect the DNA methylation at some loci[28]–[30], and *drm2* 5×L*er* (homozygous *drm2* backcrossed five times to L*er*). Methylation at *MPF* was sensitive to the deficiency in the RdDM machinery: all mutants tested, with the exception of *kyp-2*, completely relieved methylation in all three sequence contexts at *MPF* (Figure 3A and Figure S2A). Although *KYP* has been reported to control CNG methylation together with *CMT3* [26],[30], the methylation at *MPF* was independent of its function, perhaps because *MPF* at several hundred base pairs is too small for KYP to maintain the positive feed back between DNA methylation and chromatin modification [30]. Alternatively, in addition to KYP, the heterochromatic feature of this region might be redundantly controlled by other two histone H3K9 methyltransferases, SUVH5 and SUVH6 [31]. In addition, methylation of the nearby TE insertion (Figure 3B and Figure S2C) was also sensitive to *ago4-1* and *hen1-1* (Figure 3B). However, none of these mutants released all DNA methylation at *AtSN1*, a retroelement which also undergoes RdDM [26] (Figure 3C). Moreover, *AGO4* complementation [15] could not restore DNA methylation at the *MPF* in *ago4-1* (data not shown). This situation resembles the *FWA* locus whose methylation, once lost in *ddm1(decrease in DNA methylation 1)* mutant, is not recovered again even in the presence of wild type *DDM1* [32]. The *MPF* in *hen1-4*, a strong *hen1* allele in the Col background, had an identical methylation pattern to Col (Figure 1). Also, the identical methylation pattern of the miRNA deficient mutant *dcl1-9* [7] to L*er* at *MPF* (Figure S2B) ruled out the possibility that the restricted methylation at *MPF* is directed by miRNAs [33]. These observations were substantially different from prior analyses of silenced loci, at which DNA methylation was often affected in certain but never all sequence contexts by mutants in the RdDM pathway [26].

### Methylation at *MPF* Is Independent of the TE Insertion Nearby

Since *MPF* is methylated and it is near to the TE insertion in *FLC*-L*er*, it was of interest to investigate whether the methylation at *MPF* is induced by the TE. We examined the methylation status of *MPF* in several accessions that are also reported to contain transposable elements inserted in the first intron of *FLC* (Figure S3A) [19],[20]. These were tested by McrBC-PCR [34] (for Bd-0, JI-1, Stw-0, Kin-0 (CS1273), and Gr-3) and bisulfite sequencing (for Da(1)-12). Although the *MPF* is methylated in Bd-0, JI-1 and Kin-0 (CS1273), it remains unmethylated in Stw-0, Gr-3 and Da(1)-12 (Figure S3B, and data not shown for Da(1)-12) indicating that the TE insertions nearby are dispensable for the methylation at *MPF*.

A previous study using 27 Arabidopsis accessions showed that the *FLC*-TE in L*er* was also detected in Dijon-G and Di-2 (Figure S3A) but was absent in the closely related Landsberg-0 or Di-1 [18]. McrBC-PCR analysis showed that *MPF* is methylated in all four of these accessions, even in those without the *FLC*-TE insertion (Figure S3C), which further confirmed that the methylation at *MPF* is independent of the TE insertion nearby.

### Origin of *MPF*-siRNAs

**Figure 4:**
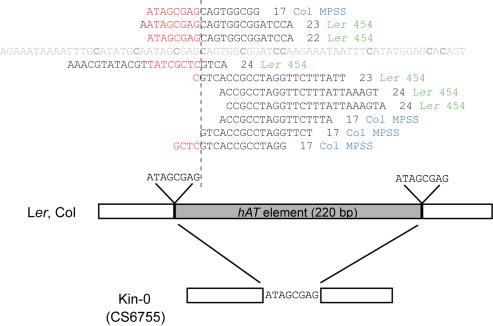
Structure of *MPF* in Three Accessions. L*er* and Col contain a 220 bp *hAT* element insertion (gray box); both the insertion and the target site duplication are absent in Kin-0. The *MPF*-siRNAs precisely match to one end of this insertion at both strands. 17 nt siRNA tags are from Col-derived MPSS dataset; the rest 22∼24 nt siRNAs sequences are from L*er*-derived 454 dataset.

To study the origin of the *MPF*-siRNAs, we found that a 220 bp sequence at *MPF* is absent in one Kin-0 accession (CS6755, different from the Kin-0 (CS1273) accession mentioned above that contains a methylated *MPF*). Further analysis revealed that this difference is caused by the insertion of a non-autonomous *hAT* element [35] with the typical 8 bp TSD (target site duplication) and short terminal inverted repeats (TIRs) (Figure 4 and Figure S1). However, *MPF*-siRNAs in L*er* are probably not derived from other *hAT* elements because those *MPF*-siRNAs with the full length information from 454 sequencing in L*er* [15] have only one match (at *MPF*) in the genome; also, genomic Southern blot hybridization revealed that L*er* do not contains extra copy of this *hAT* element comparing to Col (Figure S4). Therefore, the *MPF*-siRNAs are probably generated from *MPF* itself.

### Methylation State at *MPF* in L*er* Is Transmissible to Col by Genetic Crossing but with Extensive Diversity in the F1

**Figure 5:**
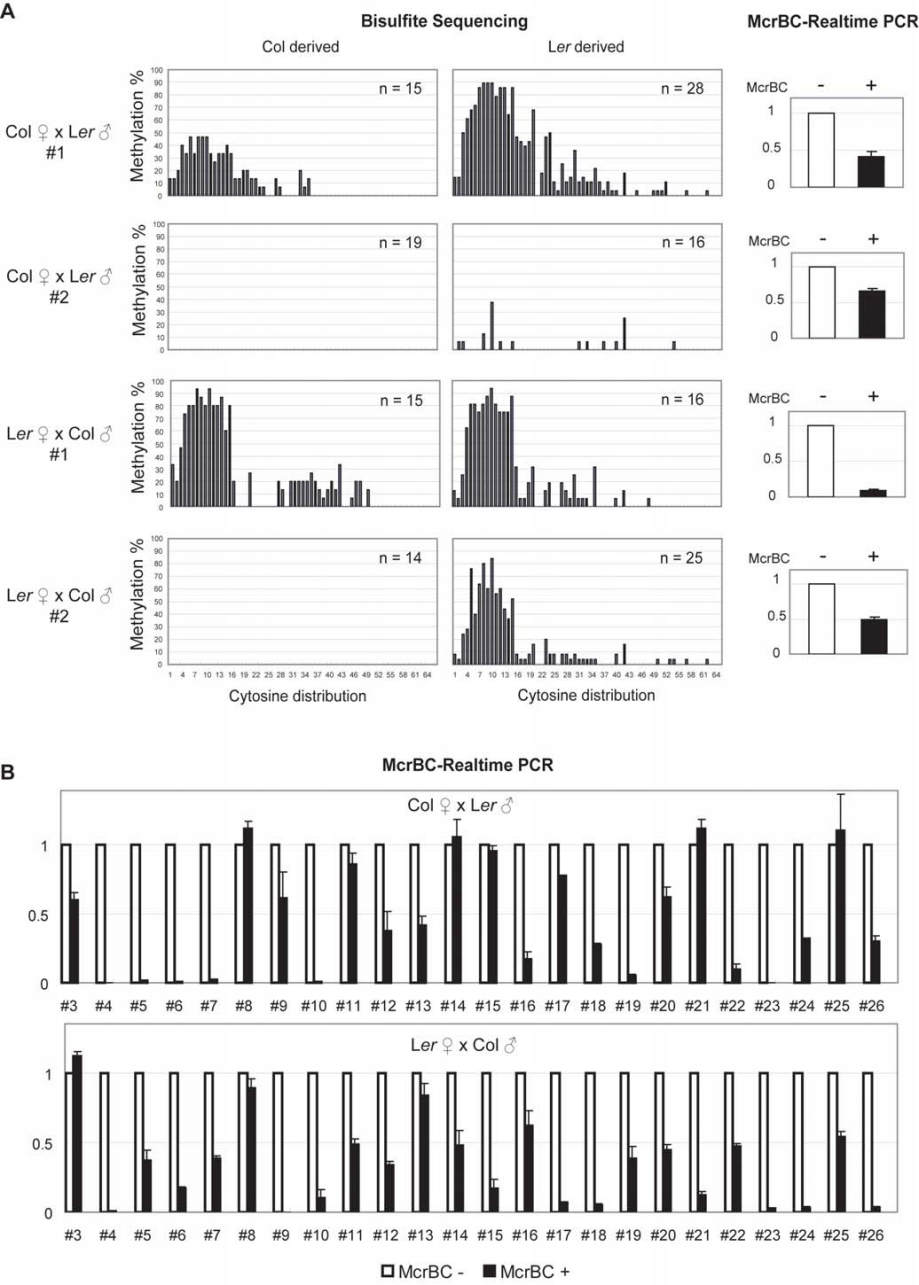
DNA Methylation Analysis in the F1 Heterozygous Plants from the Reciprocal Crosses between Col and L*er.* (A) Bisulfite sequencing analysis at *MPF* (B1 region, see Figure 2) of four heterozygous lines from both the crosses of Col♀×L*er*♂ and L*er*♀×Col♂. SNPs at *MPF* between Col and L*er* (see Figure S1) were used to distinguish the Col- and L*er*-derived sequences from the heterozygous plants. “n” indicates the number of sequenced clones. The DNA methylation status was further confirmed by real-time McrBC-PCR using the McrBC non-digested (white bar) and digested (black bar) DNA from the heterozygotes (same to the DNA samples used in bisulfite sequencing). (B) Real-time McrBC-PCR analysis in 24 more lines from each direction of the crosses to test their methylation status at *MPF.*

In paramutation, the silenced paramutagenic lines are able to confer the active state of the paramutable lines, and make them become paramutagenic [36]. To test whether the methylated state at *MPF* in L*er* is transmissible, we performed bisulfite sequencing to investigate the DNA methylation status in four F1 lines from the crosses of both Col ♀×L*er* ♂ and L*er* ♀×Col ♂, with the single nucleotide polymorphisms (SNPs) at *MPF* (Figure S1) used to distinguish the Col and L*er* derived sequencing results (Figure 5A). In addition, twenty-four more lines from reciprocal crosses were tested for their *MPF* methylation by real-time McrBC-PCR (Figure 5B). These experiments revealed extensive diversity in the methylation status of *MPF* in each individual line in the F1 generation. This diversity could be summarized in the following way: 1) in some lines, the *MPF*-siRNAs from L*er* are able to trigger the *de novo* methylation at Col-derived *MPF*; 2) in some other lines, not only the Col-derived *MPF* remains unmethylated, the L*er*-derived *MPF* could even lose its methylation; 3) there are also cases in which the L*er*-derived *MPF* remains methylated and Col-derived *MPF* remains unmethylated, just like their ancestors; therefore the *MPF* is semi-methylated in the whole plant.

### *De novo* Methylation at *MPF* Does Not Alter the Flowering Behavior of Col

The 1.2 kb *FLC*-TE, when inserted into a Col *FLC* genomic construct, is sufficient to cause reduced expression of *FLC* in the transgenic lines [19], therefore, it is unclear whether the *MPF* has any functional relevance in *FLC* expression. Interestingly, *FLC*-L*er* could strongly suppress the late flowering phenotype induced by *FRIGIDA* (*FRI*) and *luminidependens* (*ld*), but remains moderately sensitive to other mutants that up-regulate *FLC* like *fca*, *fve*, and *fpa* [37]. Recently, SUPPRESSOR OF FRI4 (SUF4) has been shown to bind to the promoter of *FLC* and directly interact with FRI and LD [38]. Moreover, *FLC*-L*er* is again sensitive to *FRI* in a *hen1-1* background [22] suggesting reversible epigenetic alteration might account for this weak response.

To address the role of the epigenetic variation at *MPF* in flowering time control, we used an RNAi approach to artificially methylate *MPF* in Col, the ecotype in which *MPF* is originally unmethylated. All transgenic plants used for further analyses had been tested for their successful *de novo* methylation at *MPF* by McrBC PCR (data not shown). Both flowering time and *FLC* expression analysis showed that *de novo* methylation at *MPF* does not alter the flowering behavior of wild type Col (Figure S5). However, since Col is an early flowering ecotype and its *FLC* expression level is relative low, we can not rule not the possibility that *MPF* may play a more prominent role in some late flowering backgrounds with higher *FLC* levels, like *FRI* or *ld*.

### Genome-Wide Identification of ∼24 nt siRNAs Directed Epigenetic Natural Variation

**Figure 6:**
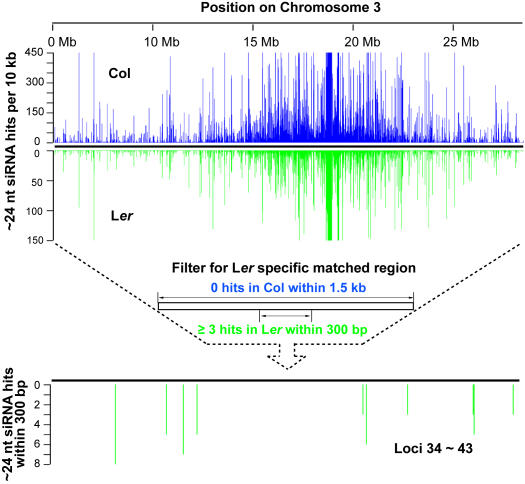
Illustration of the Strategy for Identifying Loci Matched by Significant Level of ∼24 nt siRNA Specifically in L*er* using Chromosome 3 as an Example. Unique small RNAs obtained by 454 sequencing from Col and L*er* ≥23 nt were mapped to the genome, then the perfect matches were counted per 100 bp. With this information, a filter was used to further identify loci with no less than three hits within 300 bp in L*er* versus no hits within 1500 bp for the same region in Col.

The identification of *MPF*-siRNAs in L*er-* but not Col-derived small RNA data made us wonder whether other loci are differentially and specifically matched by ∼24 nt siRNAs in these ecotypes. Because the MPSS small RNA sequencing data are not readily comparable with the 454 data (due to length differences in the sequencing reads), the small RNA datasets we used for a genome-wide identification are all 454 sequencing data, derived from two recent studies: 247,318 unique small RNA sequences from Col [16]and 25,981 unique small RNA sequences from L*er* [15]. Also, to balance the enrichment of longer siRNAs in the sequencing results of AGO4 precipitated pool from L*er* [15], we only selected for further analyses the siRNA reads of length no less than 23 nt, hence most of the miRNAs and short sRNAs are discarded from both the Col and L*er* datasets. Since only the Col genome sequence is complete and the number of sequenced Col derived siRNAs is much greater than that of L*er*, in this study, we only analyzed the regions matched by clusters of siRNAs present specifically in L*er*, to exclude the interference of genetic alteration and also for higher reliability (please see materials and methods for details about the bioinformatic analysis). The unique siRNA sequences over 23 nt from both Col and L*er* were mapped to the genome, respectively, and hits were counted in windows of 100 bp. Although the majority of the ∼24 nt small RNA clusters are conserved between Col and L*er* (data not shown), after combining the overlapping regions, 68 unique loci were identified (including the *MPF*, locus #57; Table S1). These all shared the characteristic that they were matched by at least three distinct siRNAs within 300 bp in L*er* but there were no hits in 1500 bp around the same region in Col (see Figure 6 for an example). Most of these loci are *MPF*-like, in that the siRNA matches are restricted to a small region (Figure S6), and their distribution in the genome is quite dispersed (Figure S7). Twenty-two loci are within known genes, and the other 46 are in intergenic regions (Table S2). An search of methylation data in Col (http://signal.salk.edu/cgi-bin/methylome) [25] demonstrated that all of these loci except locus #60 (located in a highly methylated region longer than several hundred kb, Table S1) were clearly lacking methylation; in addition, 28 loci contain repeat-associated sequences with one end beginning close to or within the small RNA matching region, and 15 loci had matching MPSS small RNA tags [12] (Table S1). We had also searched the website of DNA methylation information on the fourth chromosome in both L*er* and Col background (http://chromatin.cshl.edu/cgi-bin/gbrowse/epivariation/) [2]. For the 13 loci (#44∼56) we identified on the fourth chromosome, six loci are found with methylation signals in their data: five loci (#46, 49, 52, 54, 55) are found specifically methylated in L*er* as expected; one locus (#53) is methylated in both ecotypes but with a much higher methylation signal in L*er* comparing to Col. Overall, our results are well supported by the two independent studies on epigenomics and epigenetic natural variation [2],[25].

**Figure 7:**
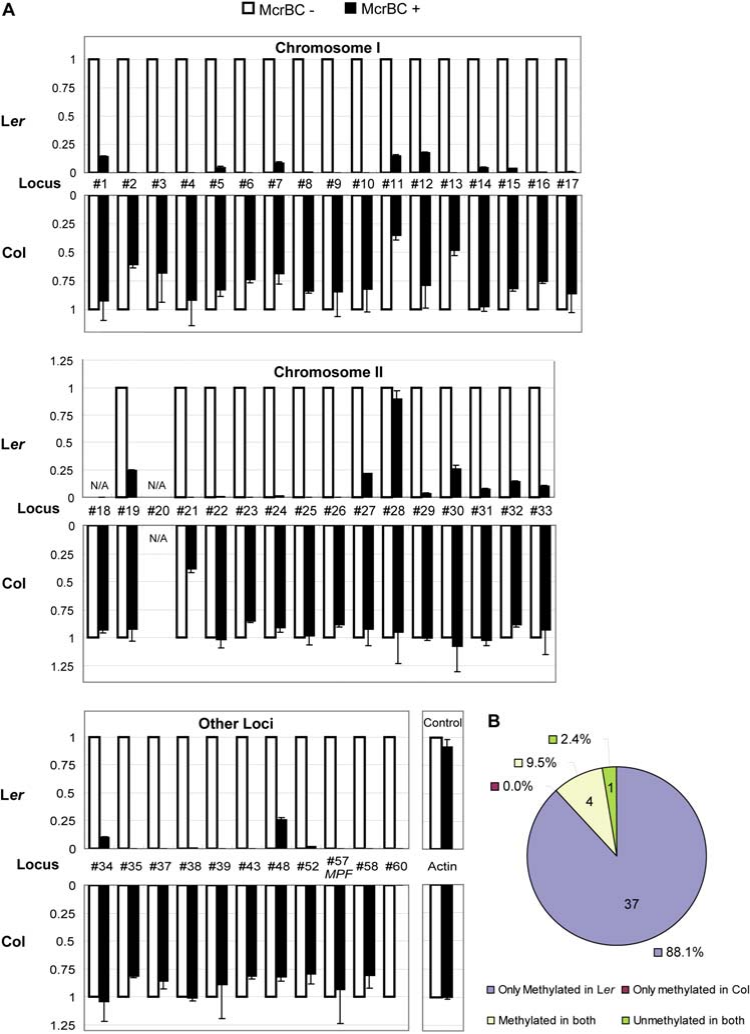
DNA Methylation Analysis of 44 loci Varied in Small RNA Abundance between Col and L*er* using Real-time McrBC-PCR. (A) Real-time PCR results using McrBC non-digested (white bar) and digested (black bar) DNA from both Col (under the axis) and L*er* (above the axis) as the PCR templates. For the comparison, the Non-digested result of each locus was normalized to 1. N/A means PCR amplification failed. If the value of McrBC digested sample at certain locus is significantly lower than McrBC non-digested one, then this locus is methylated, otherwise it is unmethylated. Locus #60 which is methylated in both Col and L*er* is used as the positive control and unmethylated *Actin* is used as the negative control. (B) Summary of the McrBC results. “Methylated” is defined as the value of McrBC non-digested sample at a certain locus is lower than 0.5, and “unmethylated” is defined as the value of McrBC non-digested sample at a certain locus is higher than 0.5.

We investigated the methylation pattern of locus #10 as an example using bisulfite sequencing. Extensive methylation was found in L*er* (Figure S8), whereas the same region in Col remained unmethylated (data not shown). Other eight randomly selected loci were tested using methylation sensitive McrBC-PCR, and all of them, even those with the minimal number of three unique siRNAs, were methylated in L*er* but not Col (Figure S9). Furthermore, we tested the methylation status of 44 loci (in which 42 have successful amplification results), including all the loci on Chromosome I and II,, by real-time McrBC-PCR (Figure 7A). From these analyses, 88% of the loci (37 out of 42) were found to be specifically methylated in L*er* but not Col, and no locus was found only methylated in Col, strongly supporting the role of ∼24 nt siRNA in triggering epigenetic natural variation (Figure 7B).

For the features of these 68 loci showing evidence of small RNA-directed variation in DNA methylation, we looked at the genes either corresponding to or adjacent to these loci within less than 1 kb distance of flanking sequence. Among the 64 genes identified (some intergenic loci did not have flanking genes within 1 kb upstream and downstream), 22 genes were found matched by genic siRNA clusters; 18 genes contained siRNA clusters in their 5′ region and 24 genes with clusters in 3′ regions (Table S2). Among the 22 genic regions, six were transposable elements, consistent with the role of transposable element in epigenetic regulation [39]. Moreover, many of these genes are reported or predicted to have important functions (Table S2). Therefore, additional investigation of these genes may help us to understand the role of epigenetic alteration in evolution and natural variation.

## Discussion

Natural variation is a fundamental aspect of biology, and the implications of natural variation for deciphering the genetics of complex agricultural traits have been widely used. Recent progress in epigenetics has revealed mechanisms that can heritably regulate gene function without alteration of primary nucleotide sequences. Although the importance of epigenetic natural variation have become more and more noticed [3],[5], the role of epigenetic regulation in evolution has been less well studied due in part to limitations in the techniques used for the investigation of epigenetic variation among natural populations. Recently, substantial improvements in high-throughput analysis approaches have made it possible for the effective detection of variation in DNA methylation, histone modifications and small RNA abundances [2], [12]–[16],[25],[40]. Small RNAs that can target DNA methylation and chromatin modifications have been proposed as a potential source in inherited epigenetic differences [3], and the latest techniques offer rapid and relatively inexpensive means for the profiling of small RNAs. In this study, we discovered that a *hAT* element adjacent to the promoter of *FLC*, which we named *MPF*, is methylated and heterochromatic in L*er* but not Col because of their differences in the abundance of corresponding siRNAs. Furthermore, by comparisons between L*er* and Col of publicly available small RNA data produced by high-throughput sequencing [15],[16], we identified at least 68 loci that are matched by significant levels of ∼24 nt siRNAs, and 88% examined loci are methylated specifically in L*er* but not Col. Our data reveal that there could be a considerable amount of small RNA-directed epigenetic natural variation between two ecotypes of *Arabidopsis*.

Although we identified dozens of loci, this analysis is still far from saturating. A *Sadhu* element (At2g10410), which was reported to be epigenetically silenced in L*er* and other 18 strains but highly expressed in Col, did not show up among the 68 loci [41]; although bisulfite sequencing revealed that this element contains CNG and asymmetric methylation in L*er*, which is presumably siRNA-directed to some extent [41]. Furthermore, hundreds of additional loci with one or two hits specifically in L*er* (data not shown) may also be silent; these may be better characterized when additional L*er* small RNA and genome sequence data become available.

Two examples of siRNA-associated, naturally-occurring epigenetic variation have been well studied in plants, including the phosphoribosylanthranilate isomerase (*PAI*) gene family in *Arabidopsis* and paramutation in maize [36]. In some *Arabidopsis* ecotypes, two *PAI* genes form an inverted repeats that may generate siRNAs and silence related members in the same gene family [42]. Paramutation, the allele-dependent transfer of heritable silencing state from one allele to another [36], is associated with another type of repeats, the tandem repeats. *MEDIATOR OF PARAMUTATION 1 (MOP1)* [43], whose deficiency disrupts paramutation, is an ortholog of the *Arabidopsis RDR2 (RNA Dependent RNA polymerase 2)*, an essential component of RNAi machinery [6]. Notably, epigenetic variation at the *MPF* is quite different from these two cases: first, neither inverted- nor tandem-repeats features were found at *MPF* or elsewhere in the genome with similar sequence; second, the level of *MPF*-siRNAs is high in L*er* and low in Col, instead of all-or-none; third, the restricted location of *MPF*-siRNAs is markedly different from the dispersed distribution of siRNAs from most inverted or tandem repeats [12].

Although paramutation phenomenon had been well documented, the details of how the silencing signal is transmitted from one allele to the other in the F1 heterozygote are still less understood. In our study, the diverse methylation status among individuals in F1 generation of the reciprocal crosses from Col×L*er* indicate that there might be a reprogramming stage shortly after fertilization, in which the DNA or chromatin are open to modifiers like the *MPF*-siRNA containing RISC (RNA induced silencing complex) from L*er*. However, this open stage must be very short, and when it is over, the epigenetic state, no matter active or silenced, will be maintained in the following developmental processes, so that the unmethylated state of Col-derived *MPF* and the methylated state of L*er*-derived *MPF* could well maintained in L*er* ♀×Col ♂line #2 (Figure S5A).

Thus far, the function of ∼24 nt siRNAs in plants has mainly been ascribed a role in silencing transposable elements and repeat-associated sequences [39]. Thus, it is unclear how L*er* and Col, both with the functional RNAi machinery, might acquire many siRNA-directed epigenetically variable loci. One characteristic of *MPF*-siRNAs, their very restricted location (all matching to a region less than 50 bp), may confer on them more flexibility than other, larger silent loci.

Genetic variability (due to insertion, deletion and point mutation) occurs stochastically, at very low frequency, primarily irreversibly and is often recessive. In contrast, heritable epigenetic variability may be more appropriate to regulate, rather than disrupt or create, gene function, and thus may be an ideal or more dynamic force for evolutionary change of gene regulation.

## Materials and Methods

### Plant Materials

The Bd-0 (CS962), JI-1 (CS1248), Stw-0 (CS1538), Gr-3 (CS1202), Kin-0 (CS1273, CS6755), Da(1)-12 (CS917), Dijon-G (CS910), Di-1 (CS1108), Di-2 (CS1110), and La-0 (CS1299) accessions of *Arabidopsis* were acquired from ABRC; *hen1-1* (L*er* background), *hen1-4* (Col background), and *dcl1-9* mutants were described before [22]; *cmt3-7*, *kyp-2*, *ago4-1*, and *drm2* 5×L*er* were generous gifts from Steve Jacobsen at UCLA. The *AGO4* complementation lines were kindly provided by Gregory J. Hannon at CSHL and Yijun Qi at NIBS.

### Small RNA Northern Blot

RNAs were extracted from 20-day-old, soil-grown plants. ^32^P end-labeled LNA probe was used for hybridization. Total RNAs were extracted using Trizol solution (Invitrogen) from 20-d-old soil-grown plants and dissolved in RNase free water. Small sized RNAs were enriched by adding the same volume of 8M LiCl and centrifuging at 12,000rpm for 30 min at 4°C. RNA filter hybridizations were carried out as previously described [44]. LNA probe [45] was used for hybridization (5′- cgagcAgtGgcGgatCcaaga-3′; uppercases represent modified nucleotides).

### Chromatin Immunoprecipitation (ChIP) Assays

The ChIP assays were performed using 20-d-old soil-grown plants and as previously described [46]. Antibodies against H3K9me1 (07-450), H3K9me2 (07-441) and H3K9me3 (07-442) were from Upstate Biotechnology.

### Construction of RNAi Vector

The genomic DNA from Col was used as a template for PCR amplification using the primer pairs (CX2004: ctcgagATTTTTGTGGTAATATATATATA and CX2005: agatctACATCAATCCAAGTTCAAGC, carrying the *Xho*I and *Bgl*II sites, respectively). The PCR products were sequentially inserted into pUCC-RNAi vector using the *Xho*I/*Bgl*II and *Bam*HI/*Sal*I sites for both the sense and antisense orientations. The stem-loop structured fragment was cut off and further cloned into a modified pCambia1302 vector (pCambia1302-LX-1) and used for plant transformation (XF718). All transgenic plants used for further analyses had been tested for their successful *de novo* methylation at *MPF*.

### DNA Methylation Analysis: Southern Blot, Bisulfite Sequencing, and McrBC-PCR

Genomic DNA was isolated from rosette leaves of 4-week-old, soil-grown plants. Southern blots was performed as previously described [22] using PCR products amplified from *FLC* promoter as the probe (Figure 1). Bisulfite sequencing experiments were performed as previously described [47]. Primers with one end in *FLC*-TE and the other in *FLC* were designed to specifically amplify the *FLC*-TE and exclude other TEs in the genome. Only the cytosines within TE were counted for methylation analysis of *FLC*-TE in Figure 3. McrBC-PCR experiments were performed as previously described [34],[47], Equal amounts of McrBC-digested and non-digested DNA were used for PCR amplification. Real-time McrBC-PCR was performed to quantitatively measure the methylation level. The primer information for these experiments could be found in Supporting Information (Text S1).

### Bioinformatics

After discarding smaller (<23 nt) and redundant sequences, 247,318 unique small RNA sequences in Col and 25,981 unique small RNA sequences in L*er* were used for further analysis. All these siRNAs were mapped to the Col genome by BLAST [48] and PERL scripts, and the numbers of perfect matches were counted per 100 bp. Next, regions contain more than 3 hits within 300 bp in L*er* but no hits in 1.5 kb at the same region in Col (Figure 6) were filtered out and overlapping regions were artificially combined. Col derived small RNA dataset was downloaded from NCBI GEO (GSE5228), and L*er* derived small RNA sequences from NCBI GenBank (DQ927324-DQ972825). The Arabidopsis genome (Col) information was provided by TIGR (release version 5). Gene positions were annotated according to TAIR's SeqViewer data. Tandem gene duplication information was provided by TIGR (tandem_gene_duplicates.Arab_R5).

## Supporting Information

Figure S1 Sequence Alignment of *MPF* Region in Col and *Ler*. Gray shades indicate the polymorphism; green box indicates the *hAT* element insertion; red region indicates the TSDs (Target Site duplication); blue region indicates the TIRs (Terminal Inverted Repeats).
(10.02 MB DOC)

Figure S2 Bisulfite Sequencing Analysis of DNA Methylation at the *MPF* in *kyp-2* (A), *dcl1-9* (B), and *FLC*-TE in *Ler* (C). The x axis represents the position of the cytosines within the sequencing region; n indicates the number of the sequenced clones. The B4 region spans the junction between TE (white box) and the first intron of *FLC* (gray box). Only the cytosines within TE were counted for methylation analysis of *FLC*-TE in Figure 3.
(9.01 MB TIF)

Figure S3 DNA Methylation Analysis of *MPF* among *Arabidopsis* Accessions using McrBC-PCR. (A) Summary of the TE insertions at the first intron of *FLC* in different ecotypes. The number under each accession represents the length of the TE insertion. (B) Accessions reported to contain transposable element inserted in the first intron of *FLC*. (C) Accessions that are closely related to *Ler*. Di-1 and La-0 do not contain the *FLC*-TE insertion. TE (methylated) and Actin (unmethylated) serve as controls for the McrBC-PCR assay.
(7.89 MB TIF)

Figure S4 Genomic Southern Blot Analysis for the Copy Number of hAT Element in Col and *Ler*. Genomic DNAs from both Col and *Ler* were digested by *EcoR* V, *Hpa* II and *Nco* I. A 160 bp region within the hAT element was PCR amplified and used as the probe for hybridization.
(13.48 MB TIF)

Figure S5 Target DNA Methylation to *MPF* in Col using RNAi Approach. (A) A diagram shows the 202 bp fragment used for the construction of the RNAi vector. (B) Flowering time analysis for the RNAi transgenic lines (T0 generation); each individual transgenic line was confirmed for their *de novo* methylation at *MPF*. (C) *FLC* expression analysis by real-time RT-PCR using the seedlings of one T2 transgenic line (homozygote for the transgene) which had been confirmed for its methylation at *MPF*.
(9.20 MB TIF)

Figure S6 Cluster Analysis. Small RNA hits were counted per 100 bp of a 1.5 kb range in *Ler* at the 68 loci identified in this study that have no less than 3 unique 24 nt siRNA matches within 300 bp (show in the central) and meanwhile no hits in a 1.5 kb region in Col (Figure 4).
(7.40 MB TIF)

Figure S7 Genome-wide Distribution of the 68 loci. Black bars represents loci with 3 to 5 hits within 300 bp; blue bars represents loci with 6 to 8 hits within 300 bp; red bars represents loci with more than 9 hits within 300 bp. Black rectangles represent the centromeric region.
(10.03 MB TIF)

Figure S8 RNA-directed DNA Methylation at Locus #10. (A) The siRNAs matched to this region. (B) Bisulfite sequencing results summarized in different sequence contexts; the x axis represents the position of the cytosines within the sequencing region; n indicates the number of the sequenced clones. The color coding of the cytosines in (A) matches the legend in (B).
(4.26 MB TIF)

Figure S9 DNA Methylation Analysis using McrBC-PCR. McrBC cuts at methylated sites in the template DNA, therefore resulting in attenuated PCR products for methylated loci; however, the PCR amplification of unmethylated loci will not be affected by McrBC digestion. (A) “Locus” represents the locus number tested from among the 68 loci that passed our filters; “hits” represents the unique siRNA hits within each 300 bp region. Locus #60 with the methylation signal in Col (Table S1) is also methylated in *Ler*. (B) The negative (*Actin*) and positive (*MPF* and *FLC*-TE) controls for McrBC-PCR. The 1.2 kb methylated *FLC*-TE is only present in *Ler*, therefore the PCR products (using primers matched to *FLC* on both sides of the TE but not within itself) from *Ler* derived samples are 1.2 kb larger than those from Col derived samples.
(6.99 MB TIF)

Table S1 The 68 Loci Identified in this Study.
(0.12 MB DOC)

Table S2 Basic Information of the Genes Corresponding or Adjacent to siRNA Clusters.
(0.11 MB DOC)

Text S1 Primer sequences.
(0.13 MB DOC)
