# Supplementary figures and images for: Small RNA-Directed Epigenetic Natural Variation in Arabidopsis thaliana
Source: PLoS Genet. 2008 Apr 25;4(4):e1000056. doi: 10.1371/journal.pgen.1000056 (PMC2289841; doi:10.1371/journal.pgen.1000056)

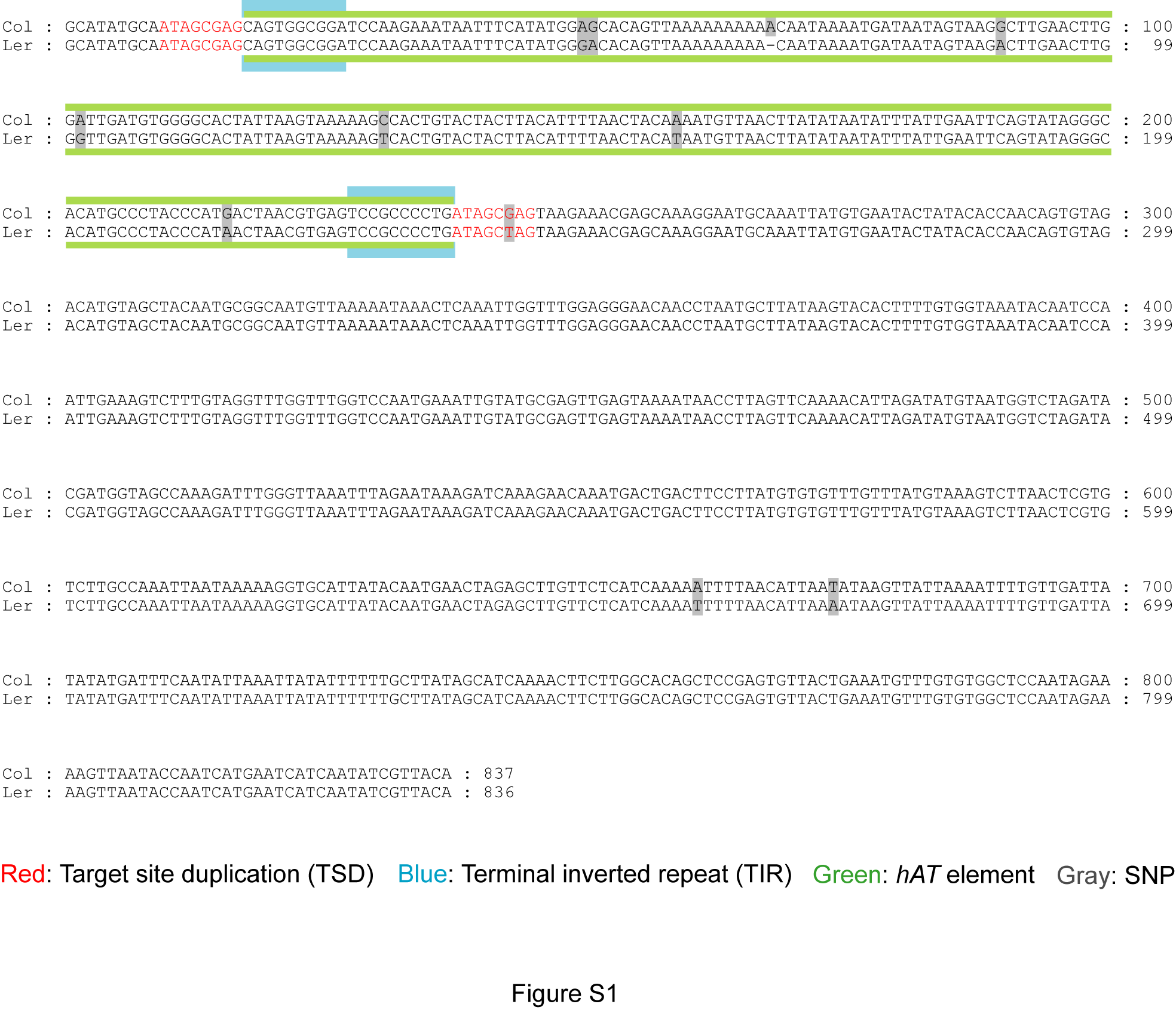

Supplement: Figure S1 — Sequence Alignment of MPF Region in Col and Ler. Gray shades indicate the polymorphism; green box indicates the hAT element insertion; red region indicates the TSDs (Target Site duplication); blue region indicates the TIRs (Terminal Inverted Repeats). (10.02 MB DOC) [file pgen.1000056.s001.tif]

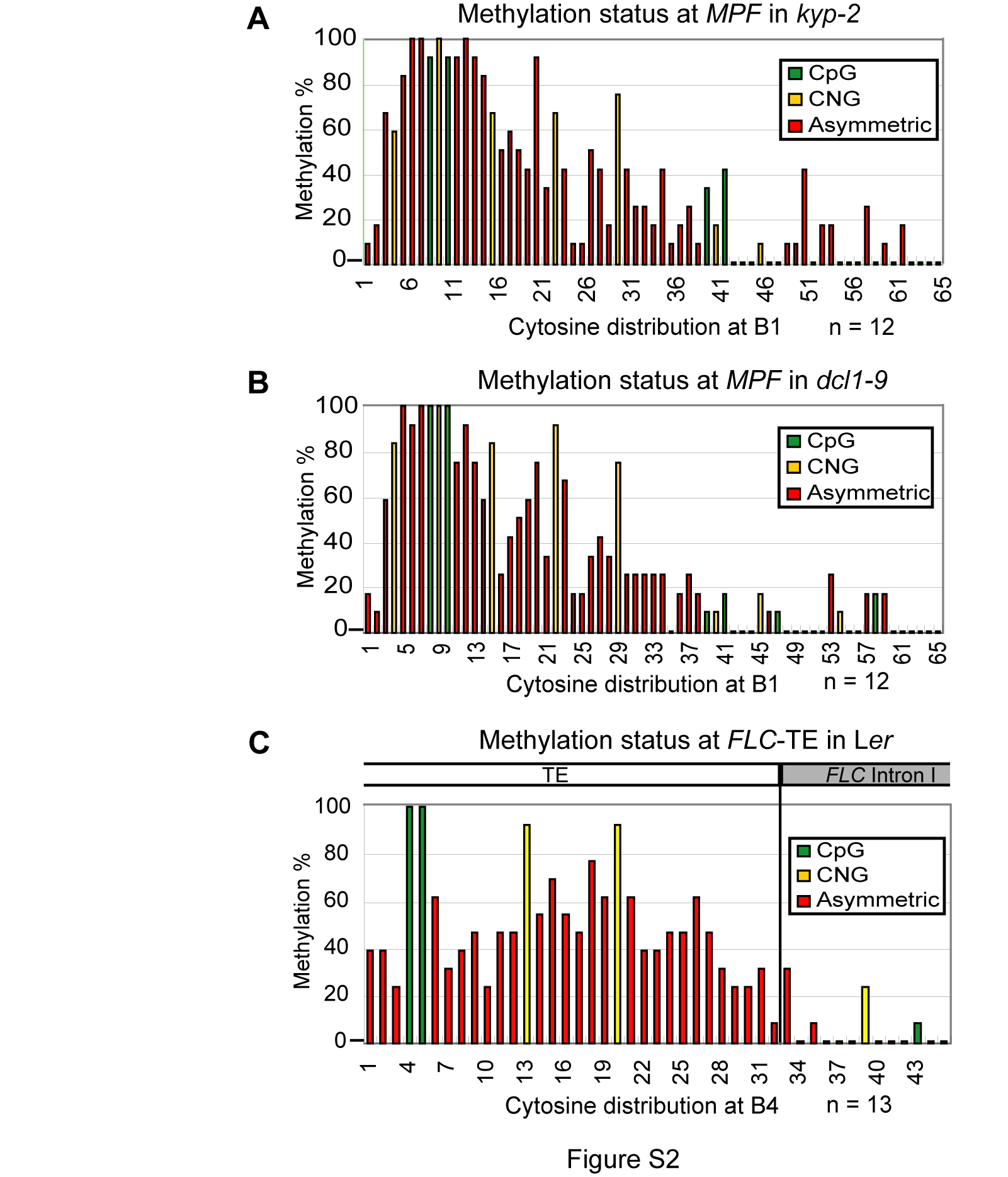

Supplement: Figure S2 — Bisulfite Sequencing Analysis of DNA Methylation at the MPF in kyp-2 (A), dcl1-9 (B), and FLC-TE in Ler (C). The x axis represents the position of the cytosines within the sequencing region; n indicates the number of the sequenced clones. The B4 region spans the junction between TE (white box) and the first intron of FLC (gray box). Only the cytosines within TE were counted for methylation analysis of FLC-TE in Figure 3. (9.01 MB TIF) [file pgen.1000056.s002.tif]

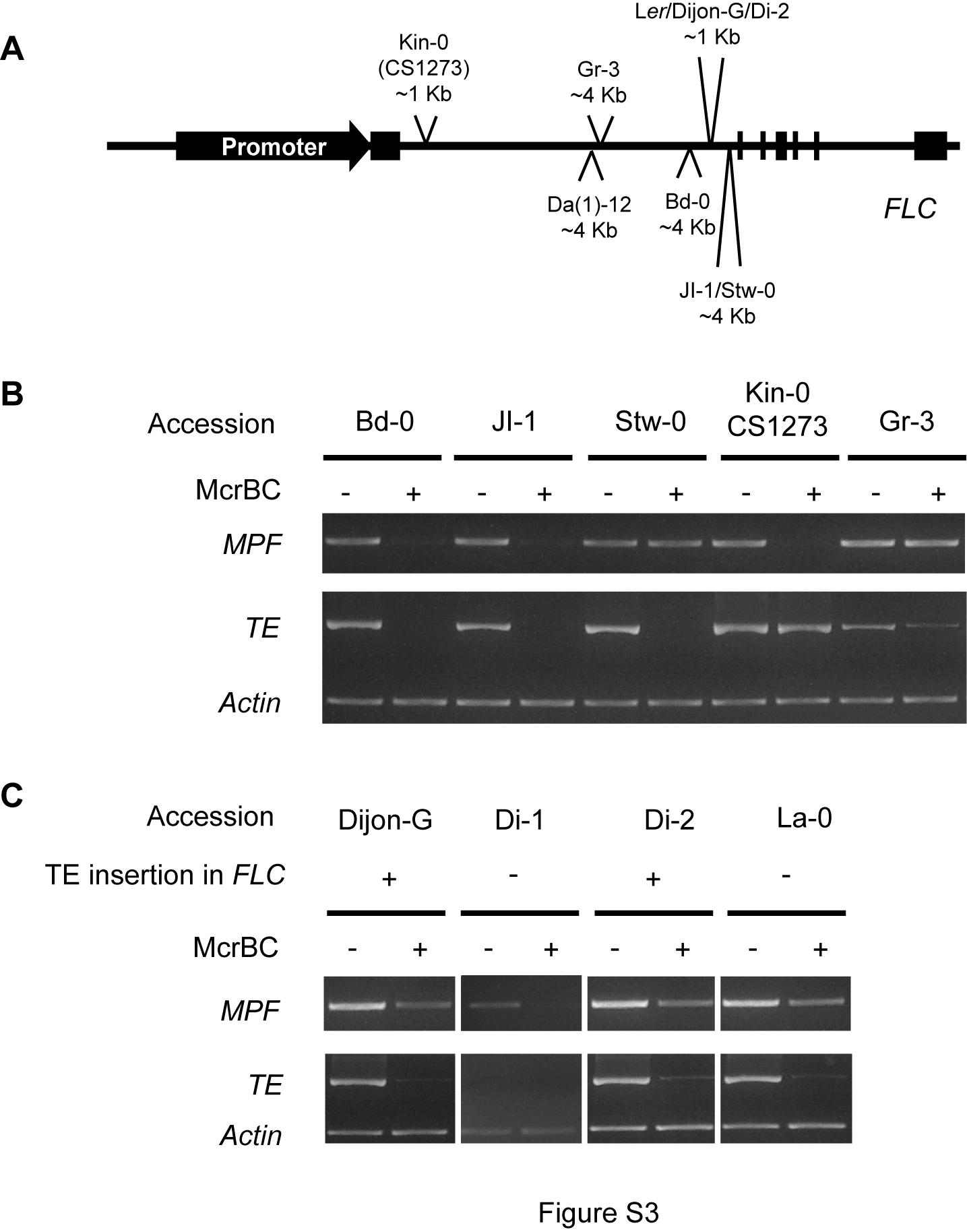

Supplement: Figure S3 — DNA Methylation Analysis of MPF among Arabidopsis Accessions using McrBC-PCR. (A) Summary of the TE insertions at the first intron of FLC in different ecotypes. The number under each accession represents the length of the TE insertion. (B) Accessions reported to contain transposable element inserted in the first intron of FLC. (C) Accessions that are closely related to Ler. Di-1 and La-0 do not contain the FLC-TE insertion. TE (methylated) and Actin (unmethylated) serve as controls for the McrBC-PCR assay. (7.89 MB TIF) [file pgen.1000056.s003.tif]

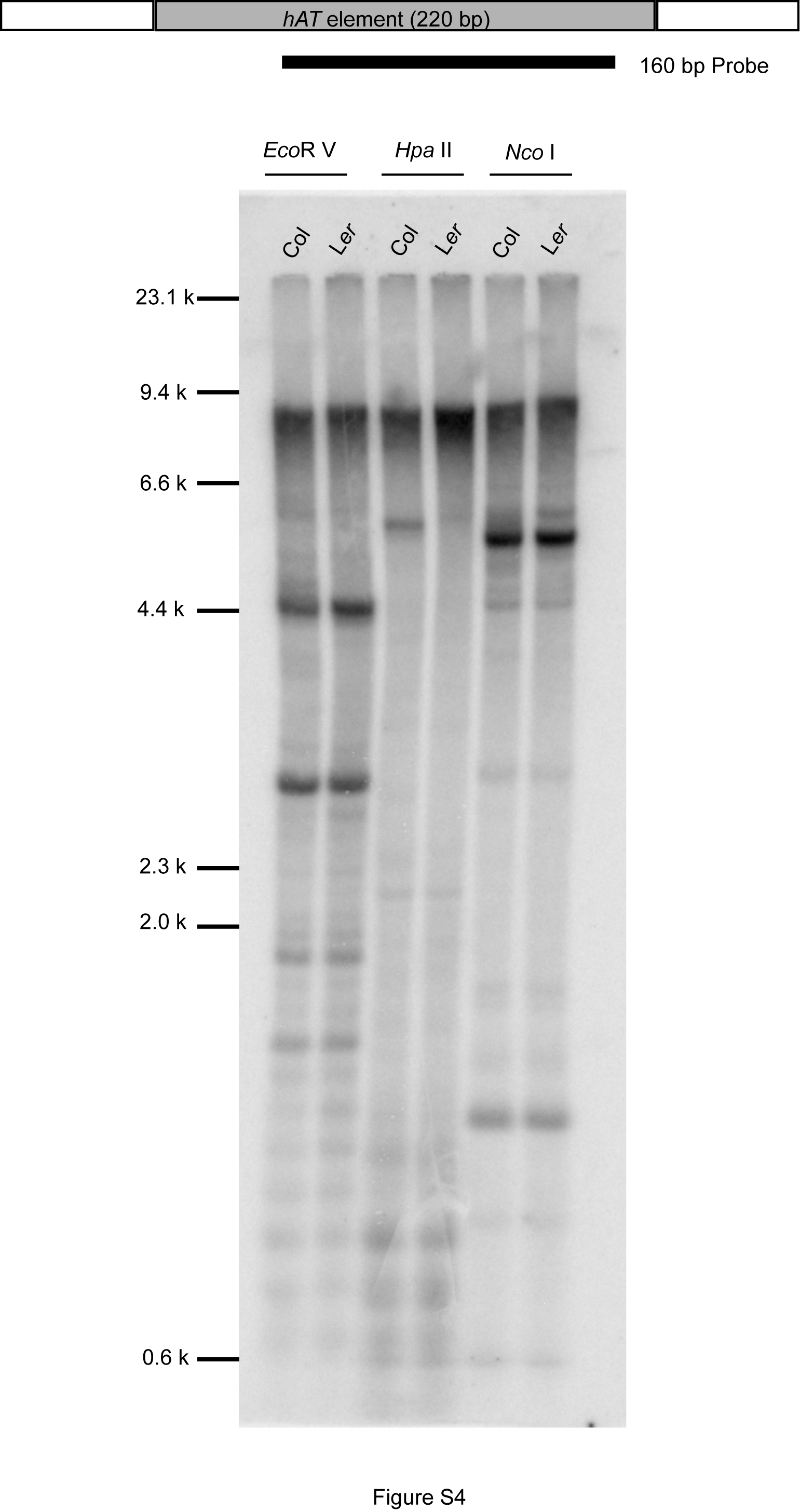

Supplement: Figure S4 — Genomic Southern Blot Analysis for the Copy Number of hAT Element in Col and Ler. Genomic DNAs from both Col and Ler were digested by EcoR V, Hpa II and Nco I. A 160 bp region within the hAT element was PCR amplified and used as the probe for hybridization. (13.48 MB TIF) [file pgen.1000056.s004.tif]

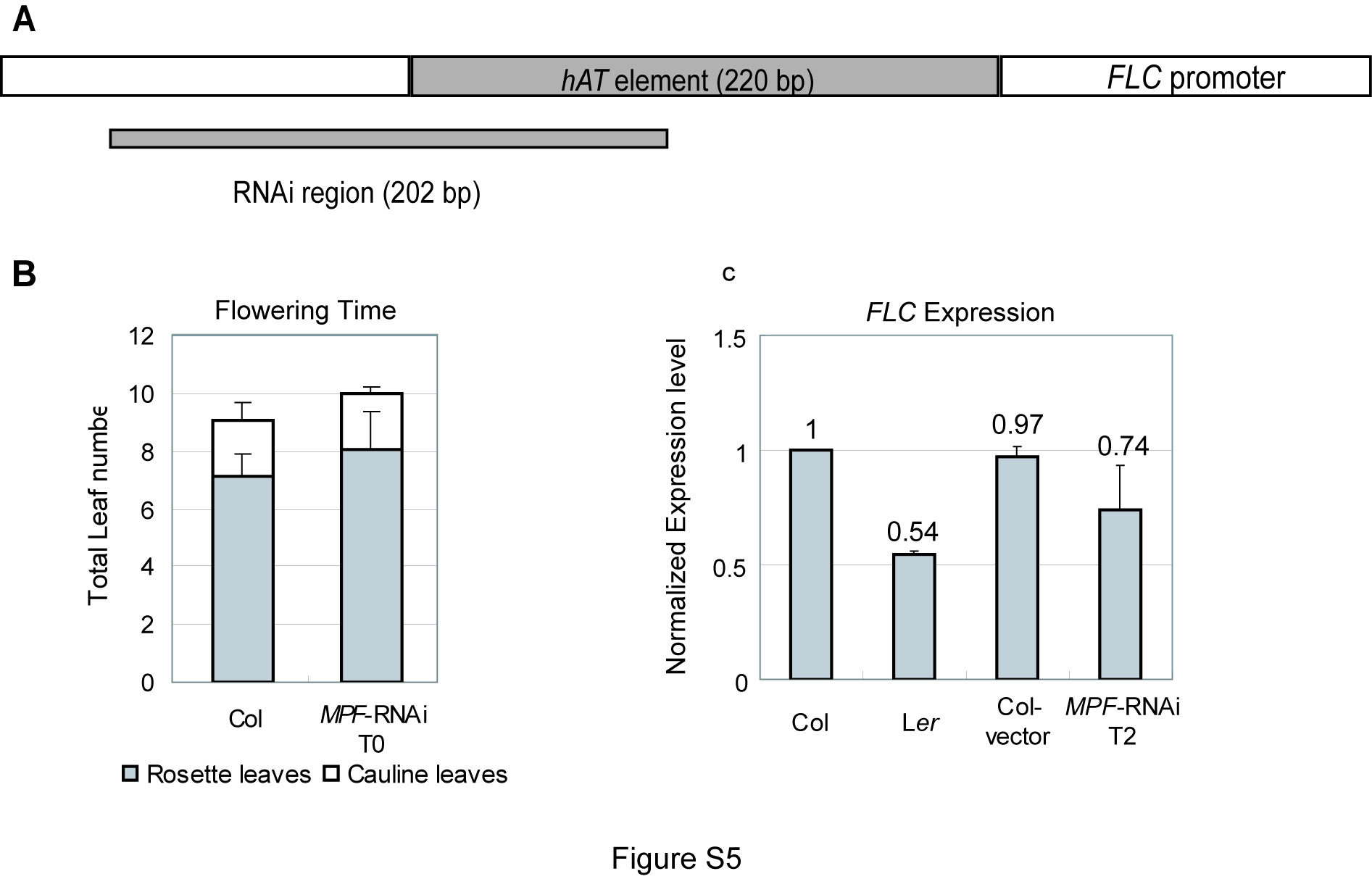

Supplement: Figure S5 — Target DNA Methylation to MPF in Col using RNAi Approach. (A) A diagram shows the 202 bp fragment used for the construction of the RNAi vector. (B) Flowering time analysis for the RNAi transgenic lines (T0 generation); each individual transgenic line was confirmed for their de novo methylation at MPF. (C) FLC expression analysis by real-time RT-PCR using the seedlings of one T2 transgenic line (homozygote for the transgene) which had been confirmed for its methylation at MPF. (9.20 MB TIF) [file pgen.1000056.s005.tif]

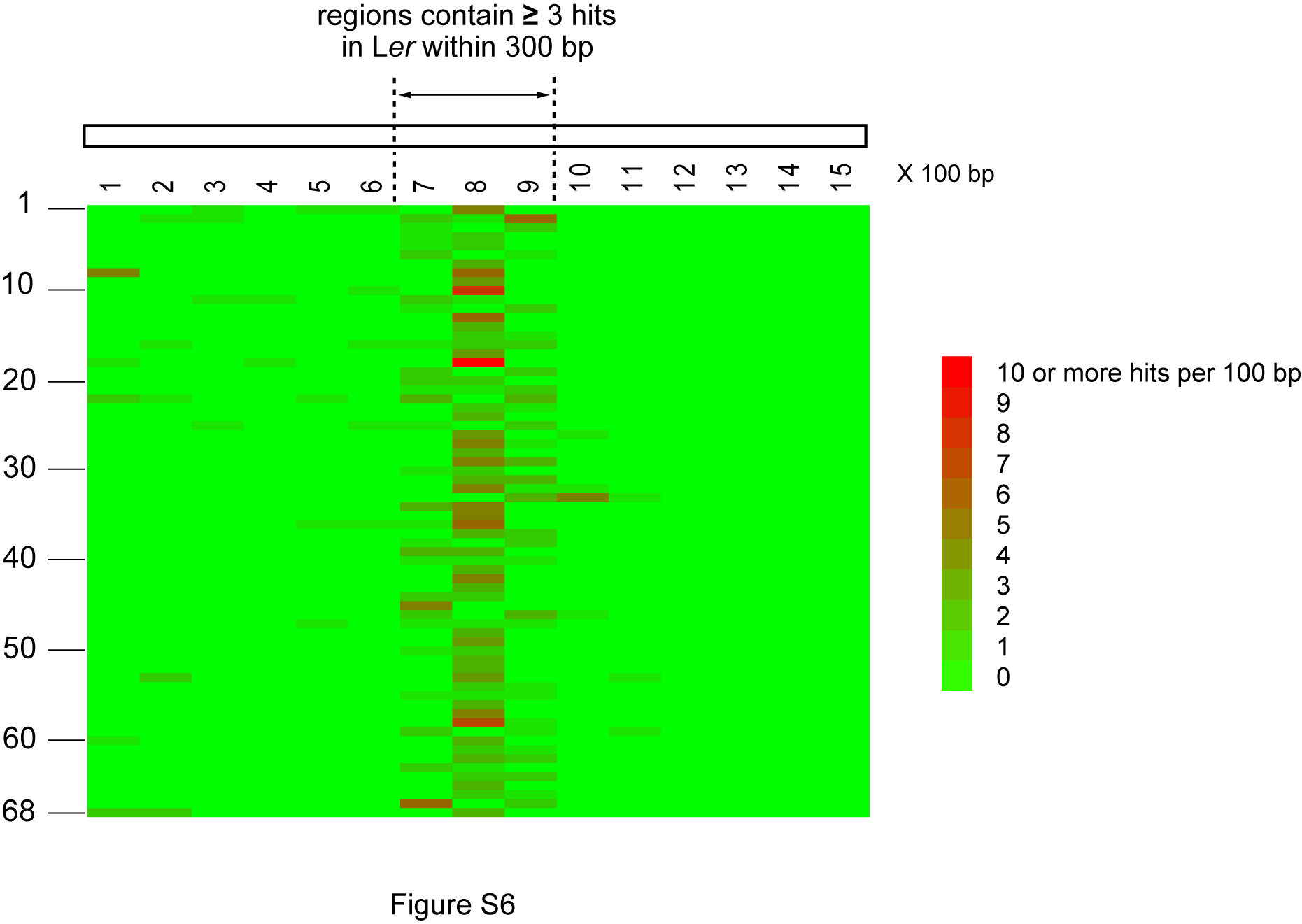

Supplement: Figure S6 — Cluster Analysis. Small RNA hits were counted per 100 bp of a 1.5 kb range in Ler at the 68 loci identified in this study that have no less than 3 unique 24 nt siRNA matches within 300 bp (show in the central) and meanwhile no hits in a 1.5 kb region in Col (Figure 4). (7.40 MB TIF) [file pgen.1000056.s006.tif]

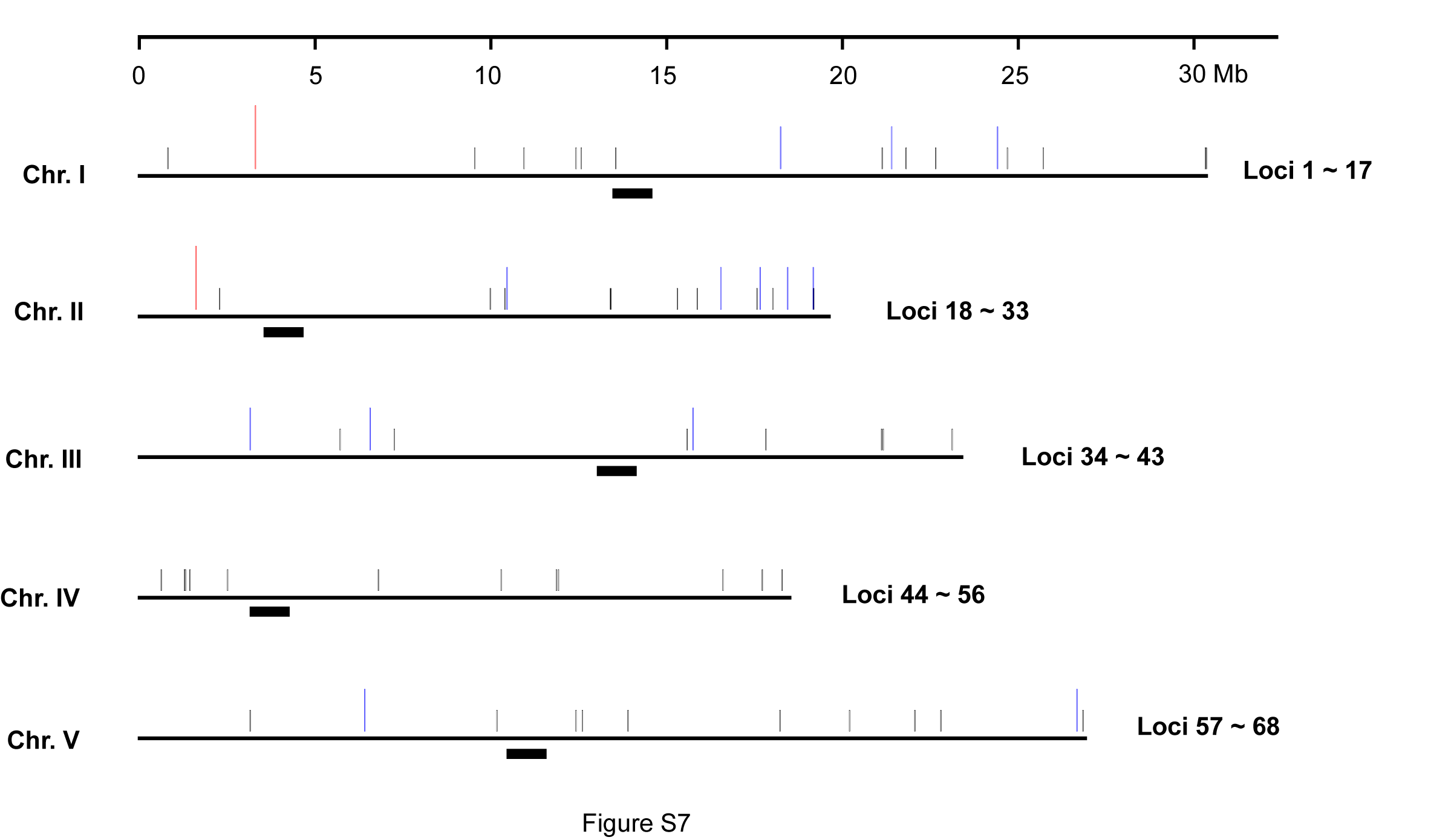

Supplement: Figure S7 — Genome-wide Distribution of the 68 loci. Black bars represents loci with 3 to 5 hits within 300 bp; blue bars represents loci with 6 to 8 hits within 300 bp; red bars represents loci with more than 9 hits within 300 bp. Black rectangles represent the centromeric region. (10.03 MB TIF) [file pgen.1000056.s007.tif]

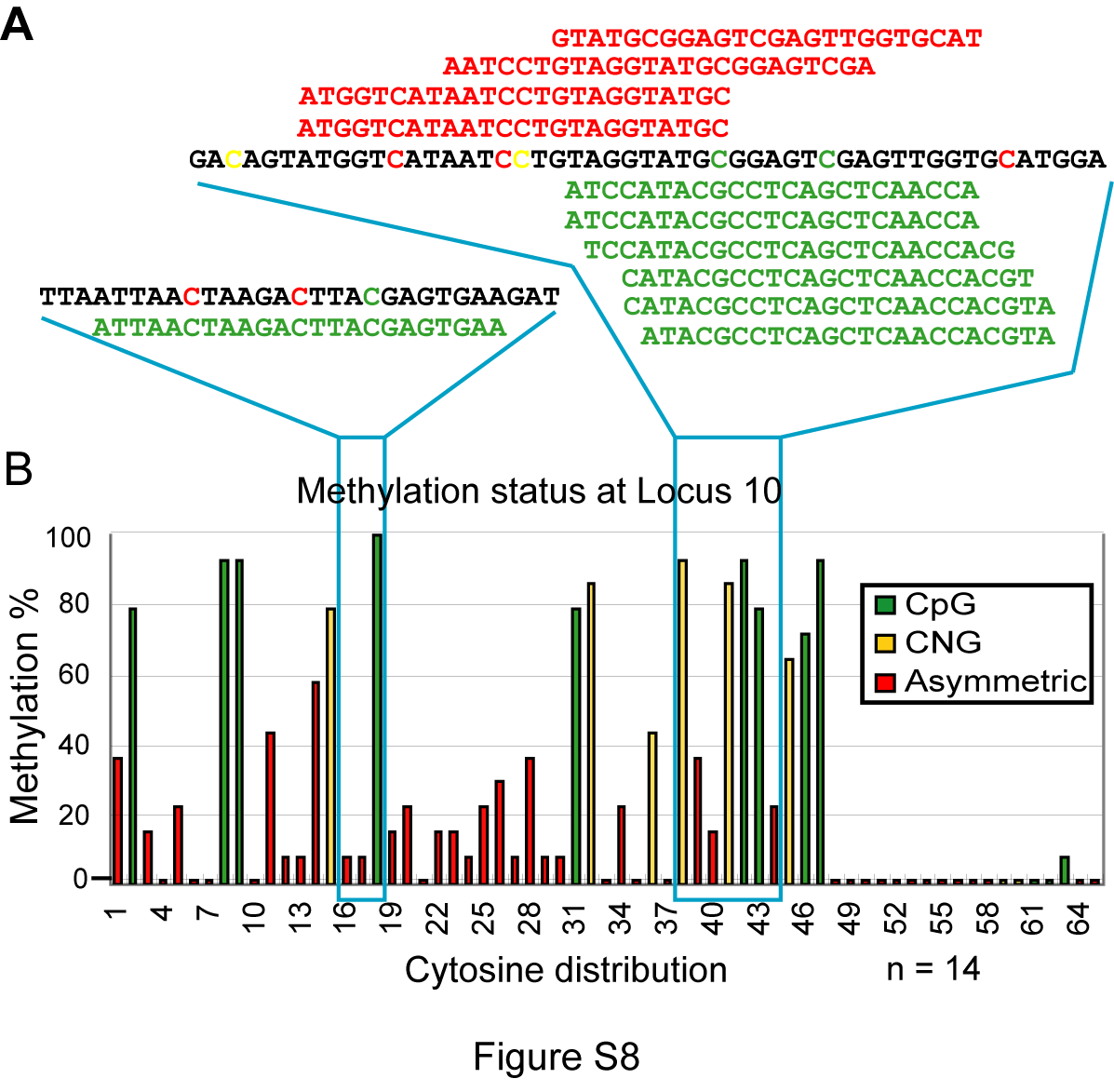

Supplement: Figure S8 — RNA-directed DNA Methylation at Locus #10. (A) The siRNAs matched to this region. (B) Bisulfite sequencing results summarized in different sequence contexts; the x axis represents the position of the cytosines within the sequencing region; n indicates the number of the sequenced clones. The color coding of the cytosines in (A) matches the legend in (B). (4.26 MB TIF) [file pgen.1000056.s008.tif]

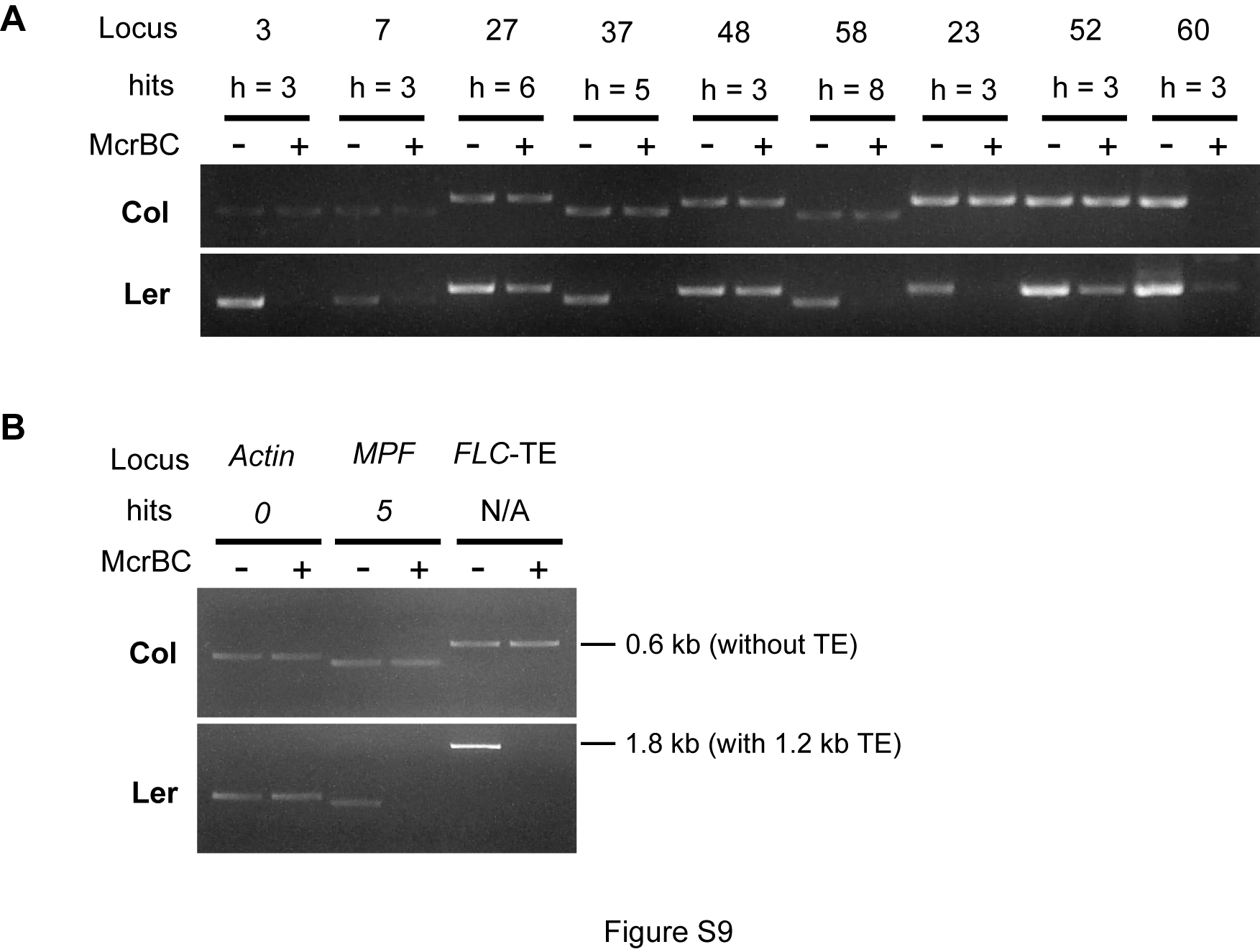

Supplement: Figure S9 — DNA Methylation Analysis using McrBC-PCR. McrBC cuts at methylated sites in the template DNA, therefore resulting in attenuated PCR products for methylated loci; however, the PCR amplification of unmethylated loci will not be affected by McrBC digestion. (A) “Locus” represents the locus number tested from among the 68 loci that passed our filters; “hits” represents the unique siRNA hits within each 300 bp region. Locus #60 with the methylation signal in Col (Table S1) is also methylated in Ler. (B) The negative (Actin) and positive (MPF and FLC-TE) controls for McrBC-PCR. The 1.2 kb methylated FLC-TE is only present in Ler, therefore the PCR products (using primers matched to FLC on both sides of the TE but not within itself) from Ler derived samples are 1.2 kb larger than those from Col derived samples. (6.99 MB TIF) [file pgen.1000056.s009.tif]
