# Supplementary material for: Small RNA-Directed Epigenetic Natural Variation in Arabidopsis thaliana
Source: PLoS Genet. 2008 Apr 25;4(4):e1000056. doi: 10.1371/journal.pgen.1000056 (PMC2289841; doi:10.1371/journal.pgen.1000056)
Supplement: Table S1 — The 68 Loci Identified in this Study. (0.12 MB DOC) [file pgen.1000056.s010.doc]

**Table S1. The 68 Loci Identified in this Study.**

| Locus | Position ^a^ | hits in L*er* ^b^ | Methylated in Col? ^c^ | Repeat associated? ^c^ | MPSS tag in Col? ^c^ |
| --- | --- | --- | --- | --- | --- |
| 1 | CHR1 - 847501~847701 | 5 | N | N | Y |
| 2 | CHR1 - 3333201~3333401 | 9 | N | N | N |
| 3 | CHR1 - 9570601~9570801 | 3 | N | Y | N |
| 4 | CHR1 - 10971501~10971701 | 3 | N | N | N |
| 5 | CHR1 - 12454901~12455101 | 3 | N | Y | N |
| 6 | CHR1 - 12602601~12602801 | 3 | N | Y | N |
| 7 | CHR1 - 13589601~13589801 | 3 | N | N | N |
| 8 | CHR1 - 18276601~18276801 | 6 | N | Y | N |
| 9 | CHR1 - 21162701~21162901 | 4 | N | Y | N |
| 10 | CHR1 - 21430601~21430801 | 8 | N | Y | N |
| 11 | CHR1 - 21841501~21841701 | 3 | N | Y | Y |
| 12 | CHR1 - 22682701~22682901 | 3 | N | Y | N |
| 13 | CHR1 - 24444901~24445101 | 6 | N | N | Y |
| 14 | CHR1 - 24725801~24726001 | 3 | N | N | N |
| 15 | CHR1 - 25749401~25749601 | 3 | N | N | N |
| 16 | CHR1 - 30351401~30351601 | 5 | N | N | Y |
| 17 | CHR1 - 30381501~30381701 | 4 | N | N | N |
| 18 | CHR2 - 1643901~1644101 | 11 | N | Y | N |
| 19 | CHR2 - 2313801~2314001 | 4 | N | Y | N |
| 20 | CHR2 - 10017501~10017701 | 4 | N | N | N |
| 21 | CHR2 - 10439401~10439601 | 4 | N | Y | N |
| 22 | CHR2 - 10496001~10496201 | 6 | N | Y | N |
| 23 | CHR2 - 13438101~13438301 | 3 | N | Y | N |
| 24 | CHR2 - 13441301~13441501 | 3 | N | Y | N |
| 25 | CHR2 - 15337001~15337201 | 3 | N | Y | Y |
| 26 | CHR2 - 15907101~15907301 | 4 | N | N | N |
| 27 | CHR2 - 16572601~16572801 | 6 | N | N | Y |
| 28 | CHR2 - 17606801~17607001 | 3 | N | Y | N |
| 29 | CHR2 - 17698101~17698301 | 8 | N | Y | N |
| 30 | CHR2 - 18057201~18057401 | 3 | N | N | N |
| 31 | CHR2 - 18472001~18472201 | 6 | N | Y | N |
| 32 | CHR2 - 19208001~19208201 | 6 | N | Y | N |
| 33 | CHR2 - 19210701~19210901 | 3 | N | Y | N |
| 34 | CHR3 - 3182401~3182601 | 8 | N | Y | N |
| 35 | CHR3 - 5748001~5748201 | 5 | N | N | N |
| 36 | CHR3 - 6595301~6595501 | 7 | N | N | Y |
| 37 | CHR3 - 7286601~7286801 | 5 | N | Y | N |
| 38 | CHR3 - 15609901~15610101 | 3 | N | N | N |
| 39 | CHR3 - 15788201~15788401 | 6 | N | Y | Y |
| 40 | CHR3 - 17856401~17856601 | 3 | N | N | Y |
| 41 | CHR3 - 21146601~21146801 | 3 | N | N | N |
| 42 | CHR3 - 21192401~21192601 | 5 | N | N | N |
| 43 | CHR3 - 23156801~23157001 | 3 | N | N | N |
| 44 | CHR4 - 651801~652001 | 4 | N | N | N |
| 45 | CHR4 - 1319801~1320001 | 5 | N | N | N |
| 46 | CHR4 - 1344201~1344401 | 5 | N | N | N |
| 47 | CHR4 - 1470701~1470901 | 3 | N | N | N |
| 48 | CHR4 - 2549101~2549301 | 3 | N | N | N |
| 49 | CHR4 - 6834901~6835101 | 4 | N | N | Y |
| 50 | CHR4 - 10327901~10328101 | 3 | N | N | N |
| 51 | CHR4 - 11891101~11891301 | 3 | N | N | N |
| 52 | CHR4 - 11959201~11959401 | 3 | N | N | N |
| 53 | CHR4 - 16637001~16637201 | 4 | N | N | N |
| 54 | CHR4 - 16638401~16638601 | 3 | N | N | N |
| 55 | CHR4 - 17756501~17756701 | 3 | N | N | N |
| 56 | CHR4 - 18314001~18314201 | 3 | N | N | N |
| 57 | CHR5 - 3181201~3181401 | 5 | N | N | Y |
| 58 | CHR5 - 6445901~6446101 | 8 | N | N | Y |
| 59 | CHR5 - 10209101~10209301 | 3 | N | N | N |
| 60 | CHR5 - 12454601~12454801 | 3 | Y | Y | N |
| 61 | CHR5 - 12637101~12637301 | 3 | N | Y | Y |
| 62 | CHR5 - 13937101~13937301 | 5 | N | N | N |
| 63 | CHR5 - 18257201~18257401 | 3 | N | Y | N |
| 64 | CHR5 - 20232501~20232701 | 4 | N | Y | Y |
| 65 | CHR5 - 22090201~22090401 | 3 | N | N | Y |
| 66 | CHR5 - 22833701~22833901 | 3 | N | Y | N |
| 67 | CHR5 - 26703001~26703201 | 8 | N | N | N |
| 68 | CHR5 - 26873601~26873801 | 3 | N | N | N |

^a^ The location of the 300 bp region that passed the filter.

^b^ The unique siRNA hits with in this 300 bp.

^c^ These values determined by searching at <http://signal.salk.edu/cgi-bin/methylome> for methylation signals, repeat features and MPSS tags, for each respective column, by querying with the sequences of these 68 loci.
