# Supplementary material for: Small RNA-Directed Epigenetic Natural Variation in Arabidopsis thaliana
Source: PLoS Genet. 2008 Apr 25;4(4):e1000056. doi: 10.1371/journal.pgen.1000056 (PMC2289841; doi:10.1371/journal.pgen.1000056)
Supplement: Table S2 — Basic Information of the Genes Corresponding or Adjacent to siRNA Clusters. (0.11 MB DOC) [file pgen.1000056.s011.doc]

**Table S2. Basic Information of the Genes Corresponding or Adjacent to siRNA Clusters**.

| locus | Type ^a^ | Gene ID | Description ^b^ |
| --- | --- | --- | --- |
| 1 | genic-of | AT1G03420 | member of Sadhu non-coding retrotransposon family |
| 2 |  | AT1G10160 | non-LTR retrotransposon family (LINE), has a 3.9e-39 P-value blast match to GB:AAA67727 reverse transcriptase (LINE-element) (Mus musculus) |
| 10 |  | AT1G57850 | Toll-Interleukin-Resistance (TIR) domain-containing protein, domain signature TIR exists, suggestive of a disease resistance protein. |
| 16 |  | AT1G80740 | chromomethylase 1 (CMT1), identical to chromomethylase GB:AAC02660 GI:2865416 from (Arabidopsis thaliana) |
| 19 |  | AT2G05980 | non-LTR retrotransposon family (LINE), has a 1.0e-42 P-value blast match to GB:NP_038607 L1 repeat, Tf subfamily, member 9 (LINE-element) (Mus musculus) |
| 21 |  | AT2G24560 | GDSL-motif lipase/hydrolase family protein, similar to family II lipase EXL3 GI:15054386 from (Arabidopsis thaliana); contains Pfam profile PF00657: GDSL-like Lipase/Acylhydrolase |
| 22 |  | AT2G24660 | copia-like retrotransposon family, has a 1.7e-166 P-value blast match to GB:BAA78424 polyprotein (Ty1_Copia-element) (Arabidopsis thaliana)gi\|4996363\|dbj\|BAA78424.1\| polyprotein (AtRE2) (Arabidopsis thaliana) (Ty1_Copia-element) |
| 23 |  | AT2G31540 | GDSL-motif lipase/hydrolase family protein, similar to family II lipases EXL3 GI:15054386, EXL1 GI:15054382, EXL2 GI:15054384 from (Arabidopsis thaliana); contains Pfam profile PF00657: GDSL-like Lipase/Acylhydrolase |
| 24 |  | AT2G31550 | GDSL-motif lipase/hydrolase family protein, similar to family II lipase EXL3 (GI:15054386), EXL1 (GI:15054382), EXL2 (GI:15054384) (Arabidopsis thaliana); contains Pfam profile PF00657: Lipase/Acylhydrolase with GDSL-like motif |
| 25 |  | AT2G36560 | DNA-binding protein-related, contains Pfam domain PF03479: Domain of unknown function (DUF296), found in AT-hook motifs Pfam:PF02178 |
| 30 |  | AT2G43470 | expressed protein |
| 32 |  | AT2G46730 | pseudogene, similar to 68 kDa protein, blastp match of 68% identity and 4.7e-17 P-value to GP\|7271113\|emb\|CAB81547.1\|\|AJ276420 68 kDa protein (Cicer arietinum) |
| 38 |  | AT3G43690 | copia-like retrotransposon family protein, has a 1.4e-29 P-value blast match to gb\|AAG52950.1\| putative envelope protein (Endovir1-1) (Arabidopsis thaliana) (Ty1_Copia-family) |
| 45 |  | AT4G02980 | auxin-binding protein 1 (ABP1), involved in cell elongation and cell division |
| 46 |  | AT4G03050 | AOP3, encodes a 2-oxoglutarate-dependent dioxygenase that catalyzes the conversion of methylsulfinylalkyl glucosinolates to hydroxyalkyl glucosinolates. involved in glucosinolate biosynthesis and secondary metabolism |
| 48 |  | AT4G04985 | expressed protein |
| 52 |  | AT4G22760 | pentatricopeptide (PPR) repeat-containing protein, contains Pfam profile PF01535: PPR repeat |
| 54 |  | AT4G34930 | 1-phosphatidylinositol phosphodiesterase-related, contains weak similarity to 1-phosphatidylinositol phosphodiesterase precursor (EC 4.6.1.13) (Phosphatidylinositol diacylglycerol-lyase) (Phosphatidylinositol- specific phospholipase C) (PI-PLC). (Swiss-Prot:P34024) (Listeria monocytogenes) |
| 58 |  | AT5G19170 | expressed protein |
| 59 |  | AT5G28237 | tryptophan synthase, beta subunit, putative, similar to SP\|P14671 Tryptophan synthase beta chain 1, chloroplast precursor (EC 4.2.1.20) (Arabidopsis thaliana); contains Pfam profile PF00291: Pyridoxal-phosphate dependent enzyme |
| 61 |  | AT5G33382 | copia-like retrotransposon family, has a 2.3e-126 P-value blast match to GB:AAB82754 retrofit (TY1_Copia-element) (Oryza longistaminata) |
| 68 |  | AT5G67310 | CYTOCHROME P450, FAMILY 81, SUBFAMILY G, POLYPEPTIDE 1 |
| 1 | 5-prime-of | AT1G03430 | Encodes AHP5, one of the six Arabidopsis thaliana histidine phosphotransfer proteins (AHPs). AHPs function as redundant positive regulators of cytokinin signaling. Members of the AHP gene family include: AT3G21510 (AHP1), AT3G29350 (APH2), AT5G39340 (APH3), AT3G16360 (APH4), AT1G03430 (APH5) and AT1G80100 (APH6). |
| 5 |  | AT1G34200 | oxidoreductase family protein, similar to AX110P (Daucus carota) GI:285739; contains Pfam profiles PF01408: Oxidoreductase family NAD-binding Rossmann fold, PF02894: Oxidoreductase family C-terminal alpha/beta domain |
| 9 |  | AT1G56500 | haloacid dehalogenase-like hydrolase family protein, low similarity to SP\|P95649 CbbY protein (Rhodobacter sphaeroides); contains InterPro accession IPR005834: Haloacid dehalogenase-like hydrolase |
| 11 |  | AT1G59265 | copia-like retrotransposon family, has a 0. P-value blast match to dbj\|BAA78425.1\| polyprotein (Arabidopsis thaliana) (AtRE1) (Ty1_Copia-element) |
| 13 |  | AT1G65720 | expressed protein |
| 15 |  | AT1G68570 | proton-dependent oligopeptide transport (POT) family protein, contains Pfam profile: PF00854 POT family |
| 18 |  | AT2G04690 | cellular repressor of E1A-stimulated genes (CREG) family, contains 1 transmembrane domain; similar to CREG2 (GI:24371079) (Homo sapiens) and (GI:24371081) (Mus musculus); similar to cellular repressor of E1A-stimulated genes CREG (GI:3550343) (Homo sapiens) |
| 26 |  | AT2G37990 | ribosome biogenesis regulatory protein (RRS1) family protein, contains Pfam profile PF04939: Ribosome biogenesis regulatory protein (RRS1); similar to Ribosome biogenesis regulatory protein homolog (Swiss-Prot:Q15050) (Homo sapiens) |
| 33 |  | AT2G46735 | expressed protein |
| 35 |  | AT3G16850 | glycoside hydrolase family 28 protein / polygalacturonase (pectinase) family protein, weak similarity to SP\|P05117 Polygalacturonase 2A precursor (EC 3.2.1.15) (Pectinase) (Lycopersicon esculentum); contains PF00295: Glycosyl hydrolases family 28 |
| 36 |  | AT3G19080 | SWIB complex BAF60b domain-containing protein, contains Pfam profile PF02201: BAF60b domain of the SWIB complex |
| 37 |  | AT3G20830 | protein kinase family protein, contains protein kinase domain, Pfam:PF00069 |
| 43 |  | AT3G62580 | expressed protein |
| 47 |  | AT4G03340 | glycosyltransferase family 14 protein / core-2/I-branching enzyme family protein, contains Pfam profile: PF02485 Core-2/I-Branching enzyme |
| 51 |  | AT4G22580 | exostosin family protein, contains Pfam profile: PF03016 Exostosin family |
| 63 |  | AT5G45120 | aspartyl protease family protein, contains Pfam profile: PF00026 eukaryotic aspartyl protease |
| 64 |  | AT5G49760 | leucine-rich repeat family protein / protein kinase family protein, contains Pfam domains PF00560: Leucine Rich Repeat and PF00069: Protein kinase domain |
| 68 |  | AT5G67320 | WD-40 repeat family protein; similar to transducin family protein / WD-40 repeat family protein [Arabidopsis thaliana] (TAIR:AT3G49660.1); similar to putative WD-40 repeat protein family [Oryza sativa (japonica cultivar-group)] (GB:BAC84349.1); similar to Os07g0405100 [Oryza sativa (japonica cultivar-group)] (GB:NP_001059429.1); contains InterPro domain LisH; (InterPro:IPR013720); contains InterPro domain WD-40 repeat; (InterPro:IPR001680); contains InterPro domain WD40-like; (InterPro:IPR011046); contains InterPro domain Lissencephaly type-1-like homology motif; (InterPro:IPR006594) |
| 2 | 3-prime-of | AT1G10170 | NF-X1 type zinc finger family protein; similar to transcription factor [Arabidopsis thaliana] (TAIR:AT5G05660.1); similar to putative TF-like protein [Oryza sativa (japonica cultivar-group)] (GB:BAD46154.1); similar to TF-like protein [Oryza sativa (japonica cultivar-group)] (GB:AAO72621.1); similar to Zinc finger, NF-X1-type; Single-stranded nucleic acid binding R3H; Zinc finger, RING-type; Zinc finger, PHD-type [Medicago truncatula] (GB:ABE80186.1); contains InterPro domain Zinc finger, PHD-type; (InterPro:IPR001965); contains InterPro domain Zinc finger, NF-X1-type; (InterPro:IPR000967); contains InterPro domain Zinc finger, RING-type; (InterPro:IPR001841) |
| 3 |  | AT1G27560 | F-box family protein-related, similar to F-box protein family, AtFBX7 (GI:20197899) (Arabidopsis thaliana) |
| 5 |  | AT1G34190 | no apical meristem (NAM) family protein, contains Pfam PF02365: No apical meristem (NAM) protein; similar to NAM protein GI:6066595 (Petunia hybrida); nam-like protein 9 (GI:21105746) (Petunia x hybrida); NAC1 GI:7716952 (Medicago truncatula) |
| 10 |  | AT1G57840 | pseudogene, putative disease resistance protein |
| 12 |  | AT1G61470 | CCR4-NOT transcription complex protein, putative, similar to SWISS-PROT:Q60809 CCR4-NOT transcription complex, subunit 7 (CCR4-associated factor 1, (CAF1) (Mus musculus) |
| 14 |  | AT1G66310 | F-box family protein, contains F-box domain Pfam:PF00646 |
| 20 |  | AT2G23510 | transferase family protein, low similarity to EIG-I24 from Nicotiana tabacum (gi:10798748), 10-deacetylbaccatin III-10-O-acetyl transferase from Taxus cuspidata (gi:6746554); contains Pfam transferase family domain PF02458 |
| 23 |  | AT2G31530 | EMB2289/SCY2 (EMBRYO DEFECTIVE 2289, SECY HOMOLOG 2); protein translocase; similar to SCY1 (SECY HOMOLOG 1), protein translocase [Arabidopsis thaliana] (TAIR:AT2G18710.1); similar to Os05g0397700 [Oryza sativa (japonica cultivar-group)] (GB:NP_001055468.1); similar to unknown protein [Oryza sativa (japonica cultivar-group)] (GB:AAT07645.1); contains InterPro domain SecY protein; (InterPro:IPR002208) |
| 28 |  | AT2G42245 | RNA-binding protein-related, similar to mec-8 (Caenorhabditis elegans) GI:1370048 |
| 28 |  | AT2G42250 | CYP712A, CYTOCHROME P450, FAMILY 712, SUBFAMILY A, POLYPEPTIDE 1 |
| 29 |  | AT2G42490 | copper amine oxidase, putative, similar to copper methylamine oxidase precursor (MAOXII) (Arthrobacter sp.) SWISS-PROT: Q07123 |
| 32 |  | AT2G46720 | mutant has Increased stomatal density in high CO2; 3-Keto Acyl Coenzyme A Synthase; Long-Chain Fatty Acid Biosynthesis |
| 34 |  | AT3G10280 | fatty acid elongase 3-ketoacyl-CoA synthase, putative, similar to fatty acid elongase 3-ketoacyl-CoA synthase 1 GB:AAC99312 (Arabidopsis thaliana |
| 34 |  | AT3G10290 | phosphate translocator-related, low similarity to SP\|P52178 Triose phosphate/phosphate translocator, non-green plastid, chloroplast precursor (CTPT) (Brassica oleracea), phosphoenolpyruvate/phosphate translocator precursor (Mesembryanthemum crystallinum) GI:9295275 |
| 36 |  | AT3G19070 | cell wall protein-related, similar to vegetative cell wall protein gp1 (Chlamydomonas reinhardtii) gi\|12018147\|gb\|AAG45420; |
| 39 |  | AT3G43970 | expressed protein |
| 41 |  | AT3G57110 | hypothetical protein |
| 42 |  | AT3G57230 | MADS-box transcription factor. Expressed in leaf, root and stem, with higher RNA accumulation in guard cells and trichomes. |
| 42 |  | AT3G57240 | BG3, encodes a member of glycosyl hydrolase family 17 |
| 45 |  | AT4G02970 | Signal recognition particle. Type 4 of RNA polymerase III dependent genes |
| 53 |  | AT4G34920 | 1-phosphatidylinositol phosphodiesterase-related, contains weak similarity to 1-phosphatidylinositol phosphodiesterase precursor (EC 3.1.4.10) (Phosphatidylinositol-specific phospholipase C) (PI-PLC). (Swiss-Prot:P34024) (Listeria monocytogenes) |
| 57 |  | AT5G10150 | expressed protein |
| 65 |  | AT5G54360 | zinc finger (C2H2 type) family protein-related, contains Prosite:PS00028: Zinc finger, C2H2 type, domain. |
| 66 |  | AT5G56330 | carbonic anhydrase family protein, contains proline-rich extensin domains, INTERPRO:IPR002965; contains Pfam profile PF00194: Eukaryotic-type carbonic anhydrase |

^a^ Type indicates the relationship between the siRNA clusters and their nearby genes. Clusters could be at genic (blue), 5 prime (green), or 3 prime (red) of nearby genes with shorter than 1kb distance.

^b^ Gene descriptions are annotated by TAIR.
