# Supplementary material for: Small RNA-Directed Epigenetic Natural Variation in Arabidopsis thaliana
Source: PLoS Genet. 2008 Apr 25;4(4):e1000056. doi: 10.1371/journal.pgen.1000056 (PMC2289841; doi:10.1371/journal.pgen.1000056)
Supplement: Text S1 — Primer sequences. (0.13 MB DOC) [file pgen.1000056.s012.doc]

**Supporting Information**

**Primer Information (f – forward, r – reverse):**

**1. Primers for Bisulfite Sequencing at *FLC* (changed nucleotides from C to T or G to A are shown as lowercases).**

B1 f (CX1582): TTTGAAAGTttATGAAAATtATTTGGTTAGTAtTtAAT
B1 r (CX1584): AATTaaATTaTATTTACCACAAAAaTaTACTTATAA

B2 f (CX1635): GTGTTGtAAAATtGTAAATTtAATtTAATtTTATGGG
B2 r (CX1637): CTTaCAAATCAaATCAATAaTTAAACAAAAaTAAAaAACATT

B3 f (CX1640): GAGTAAAATAAttTTAGTTtAAAAtATTAGATATGTAATGG
B3 r (CX1642): TTTaTTAAAAAATaTTATATTAaACACTTaAAAaaTaAaaC

B4 f (CX2051): AAAATTtAAATtATGTTAGATAAAtAAAAAAAAATTTG
B4 r (CX2053): CCACTaaAAACTATaAAACATTaAaAAAACACCTTACC

No.10 locus f (CX2352): TTGAtGttTTTGAGAGAtATGGAATtTTGTGTTTTGTAG
No.10 locus r (CX2353): CACTCTAaCaCTTCCTTCCACTCCATaATCTaATCTCCTC

**B1 degenerated primers are used for the analysis in Col x L*er* F1 heterozygous plants.**
B1 degenerate f (CX2412): TTTGAAAGTYYATGAAAATYATTTGGTYAGTAYTYAAT
B1 degenerate r (CX2413): AATTRRATTRTATTTACCACAAAARTRTACTTATAA

The BS *AtSN1* primers had been previously described [1].

**2. Primers for ChIP Assays.**

C1 f (CX1719): CTATTGATCTGATTTGCAAG
C1 r (CX1720): CTGTTGGTGTATAGTATTCACA

C2 f (CX1717): TTCATGGGCAGGATCATCAGTCAA
C2 r (CX1718): GCTTCAACATGAGTTCGGTCTGCA
The ChIP primers for C3 was previously described [2] as Region V.

C3 f (CX0743): TTGCATCACTCTCGTTTACCC
C3 r (CX0744): GCGTCACAGAGAACAGAAAGC
The ChIP primers for C2 was previously described [3] as *FLC*-B.

*Ta3* f (CX1776): GATTCTTACTGTAAAGAACATGGCATTGAGAGA
*Ta3* r (CX1777): TCCAAATTTCCTGAGGTGCTTGTAACC
The ChIP primers for *Ta3* region was described by Johnson et al. [4].

**3. Primers for Real-time McrBC-PCR.
Note: locus 18 and 20 had not been successfully amplified in our assay**

| Locus 1 | Forward | CX2806 | GATTTGCGGCAGGACTCGTA |
| --- | --- | --- | --- |
|  | Reverse | CX2807 | CGTTCCGTTGACTGATTTTG |
| Locus 2 | Forward | CX2808 | GTTTATTTTGCAGGTCCTCT |
|  | Reverse | CX2809 | GGCATGTTTGTAACGCTTTT |
| Locus 3 | Forward | CX2543 | ACAAATATATAAGTCGGTTTAC |
|  | Reverse | CX2544 | ATAACAAAGACCATCAAACG |
| Locus 4 | Forward | CX2810 | GCCGTTTATCTTGATATGTT |
|  | Reverse | CX2811 | GTGTGAGGACACAAATTAAC |
| Locus 5 | Forward | CX2812 | CCTCATGGATGAATTATACA |
|  | Reverse | CX2813 | GGATATTCTTTAAAATCTGAACT |
| Locus 6 | Forward | CX2814 | CGTCATTAGAGAGATGAATT |
|  | Reverse | CX2815 | TGAAGAAATGGGTGATACAA |
| Locus 7 | Forward | CX2545 | CTATACAATGCTAAAATATTTCT |
|  | Reverse | CX2546 | TCTACTATTATTTCAATTTTGAA |
| Locus 8 | Forward | CX2816 | AGAAGCCTTTCGCATGAGAC |
|  | Reverse | CX2817 | TTCTGGCATTCTTTACGTCT |
| Locus 9 | Forward | CX2818 | CCTTCAAATTGATTTCCTCT |
|  | Reverse | CX2819 | TCGTGATCTCTTTGAGGATG |
| Locus 10 | Forward | CX2820 | CGCACTAGTTATCTTCTCTAG |
|  | Reverse | CX2821 | CTTCCACTCCATGATCTGAT |
| Locus 11 | Forward | CX2822 | CGAAGAAGAAATAAGAAGATCG |
|  | Reverse | CX2823 | ACAAACCAAGCAAGATCAGC |
| Locus 12 | Forward | CX2824 | GTTACATCCCGATACCGGTC |
|  | Reverse | CX2825 | CTCAAGTCCACAGGAGAAAA |
| Locus 13 | Forward | CX2826 | GTCCCTTTTATCTTCTTTTC |
|  | Reverse | CX2827 | TTCTCTAACCATAATAATTAGG |
| Locus 14 | Forward | CX2828 | GAGCATCGACATTGGTCATACC |
|  | Reverse | CX2829 | CGGCTCTCACCCATGGAAAC |
| Locus 15 | Forward | CX2830 | CGTACTACACGGACTACTTAAT |
|  | Reverse | CX2831 | GCGCTTGTTTGTGAATTATT |
| Locus 16 | Forward | CX2832 | GGTTGATGCAGTGTGTAATC |
|  | Reverse | CX2833 | ATCATGACGACTTGATGGAG |
| Locus 17 | Forward | CX2834 | CAACACCAATCTTTCTTGTT |
|  | Reverse | CX2835 | CGTGTAAAAAAGTCAGATACCT |
| Locus 18 | Forward | cx3151 | AAGTCTAAATCAGTTTATT |
|  | Reverse | cx3152 | CAAGTTTTTTGTTCATTC |
| Locus 19 | Forward | cx3153 | TTGAAAACGAGATCTTCCTAGC |
|  | Reverse | cx3154 | TGAGGAGTTGGAGACGAGG |
| Locus 20 | Forward | cx3155 | TTAAGAGATTACATTTAGTT |
|  | Reverse | cx3156 | CTTTTTCTTTTGAAATCT |
| Locus 21 | Forward | cx3157 | GTTTAGGGTTCCCACTCGTC |
|  | Reverse | cx3158 | CGTATTTGCTAGGGTTTTGC |
| Locus 22 | Forward | cx3159 | AGCTTCGGCGGAAGAGTGA |
|  | Reverse | cx3160 | CGTTTGCTTTATCTCCTTCATACT |
| Locus 23 | Forward | CX2572 | CCACACACACGAATCATATA |
|  | Reverse | CX2573 | TAGGGTTTTGGAACAGACTA |
| Locus 24 | Forward | cx3161 | GACATCGGCGTAAAGGAATT |
|  | Reverse | cx3162 | TTACCACAATTAGGGTTTTGGA |
| Locus 25 | Forward | cx3163 | GAGCAACCATAGCTTTGGAC |
|  | Reverse | cx3164 | TTAATAAGTTGTCTTACATGGATAAG |
| Locus 26 | Forward | cx3165 | CCTCACACACATATTCACCA |
|  | Reverse | cx3166 | CGTTGTTAAGAATACATTAATACCCTAT |
| Locus 27 | Forward | CX2566 | GTCAATTCTGAATAAATGCG |
|  | Reverse | CX2567 | GTTGATTCACCAATGTTACT |
| Locus 28 | Forward | cx3167 | AGAAAAAGAGGCACGCGAGG |
|  | Reverse | cx3168 | CCGCTAGATGTTACCTGTTTCTGT |
| Locus 29 | Forward | cx3169 | GGAGTTGGATCAATTGTGGAAG |
|  | Reverse | cx3170 | TGGAAGAGAGGAGCAACATCTC |
| Locus 30 | Forward | cx3171 | GAAATGATGATTATGTTACT |
|  | Reverse | cx3172 | AACAGATATGAAAATATTCA |
| Locus 31 | Forward | cx3173 | ATTCAAATTGACCCTTAAACGGC |
|  | Reverse | cx3174 | GAAATCACACACTCGAGGCTT |
| Locus 32 | Forward | cx3175 | GCTGCTTATGCAACAGGTAACT |
|  | Reverse | cx3176 | AAGGGAGGAGCAACATCTCTAC |
| Locus 33 | Forward | cx3177 | CAAGTTAAAAAAGCTTCGAACATA |
|  | Reverse | cx3178 | CCCACCAAATGGCTTGTTTT |
| Locus 34 | Forward | cx3179 | CAATCTGGTTCGTCGGAGTT |
|  | Reverse | cx3180 | TACCCCACTCTCACCTCTCG |
| Locus 35 | Forward | cx3181 | AGTTTTAGTTATCTTTTCCC |
|  | Reverse | cx3182 | GGGAATAACTTTGTTTTCATGTTT |
| Locus 37 | Forward | CX2549 | CCACCTAATCTACGATAATT |
|  | Reverse | CX2550 | GCCTCTTATTGTATTCAGTT |
| Locus 38 | Forward | cx3185 | TGCTAGTGTGCCAGATGCGG |
|  | Reverse | cx3186 | CATCTCTGGCAGATTCGCAT |
| Locus 39 | Forward | cx3187 | ATAAGTTGTGTTTCCTTGTTCACC |
|  | Reverse | cx3188 | CCGTGTCATTAGTCCAAGAAAG |
| Locus 43 | Forward | cx3195 | TAAAGGACGGTAAGGTACTCGA |
|  | Reverse | cx3196 | GACATGTTCAACATCTATGCTTGTA |
| Locus 48 | Forward | CX2568 | ATCCGATGTTGTACATTTGA |
|  | Reverse | CX2569 | CATGTCGACCTTGAGATGAT |
| Locus 52 | Forward | CX2574 | GCACCAAAAGTAATAATCAG |
|  | Reverse | CX2575 | TATGTTCACATGAGCCATGA |
| Locus 57 | Forward | CX1719 | CTATTGATCTGATTTGCAAG |
|  | Reverse | CX1720 | CTGTTGGTGTATAGTATTCACA |
| Locus 58 | Forward | CX2553 | TAACATCACACATGCCTTTT |
|  | Reverse | CX2554 | TAAATCATACCTGCCAACCG |
| Locus 60 | Forward | CX2576 | CGATGAAGAGAGCCCAAGGA |
|  | Reverse | CX2577 | AGAGAAGCGAGGTCTGCCAC |

Actin f (CX1518): GCCTAACTTTAGCCGTAACCATCAGTCAC
Actin r (CX1519): AGCGAACGGATCTAGAGACTCACCTTG

**4. Primers to Amplify the Probes for Southern Blots.**

**Promoter of *FLC*** *FLC*-P75: CGAGCAAAGGAATGCAAATT
*FLC*-P42: TTGTGCCTATCTACTTTTTC

***hAT* Element within *MPF*** Ty3-p1: ACAATAAAAT GATAATAGTA AGGC
Ty3-p2: CTCACGTTAGTCATGGGTAG

**Reference:**

1. Zilberman D, Cao X, Jacobsen SE (2003) ARGONAUTE4 control of locus-specific siRNA accumulation and DNA and histone methylation. Science 299: 716-719.

2. Liu J, He Y, Amasino R, Chen X (2004) siRNAs targeting an intronic transposon in the regulation of natural flowering behavior in Arabidopsis. Genes Dev 18: 2873-2878.

3. Bastow R, Mylne JS, Lister C, Lippman Z, Martienssen RA, et al. (2004) Vernalization requires epigenetic silencing of FLC by histone methylation. Nature 427: 164-167.

4. Johnson L, Cao X, Jacobsen S (2002) Interplay between two epigenetic marks. DNA methylation and histone H3 lysine 9 methylation. Curr Biol 12: 1360-1367.
